# Development and validation of a prediction model for invasive bacterial infections in febrile children at European Emergency Departments: MOFICHE, a prospective observational study

**DOI:** 10.1136/archdischild-2020-319794

**Published:** 2020-11-18

**Authors:** Nienke N Hagedoorn, Dorine Borensztajn, Ruud Gerard Nijman, Daan Nieboer, Jethro Adam Herberg, Anda Balode, Ulrich von Both, Enitan Carrol, Irini Eleftheriou, Marieke Emonts, Michiel van der Flier, Ronald de Groot, Benno Kohlmaier, Emma Lim, Ian Maconochie, Federico Martinón-Torres, Marko Pokorn, Franc Strle, Maria Tsolia, Dace Zavadska, Werner Zenz, Michael Levin, Clementien Vermont, Henriette A Moll

**Affiliations:** 1 General Paediatrics, Erasmus MC Sophia Children's Hospital, Rotterdam, Zuid-Holland, The Netherlands; 2 Section of Paediatric Infectious Diseases, Imperial College London, London, UK; 3 Public Health, Erasmus MC, Rotterdam, Zuid-Holland, The Netherlands; 4 Paediatrics, Children clinical university hospital, Rigas Stradinas Universitate, Riga, Latvia; 5 Division of Paediatric Infectious Diseases, Dr von Haunersches Kinderspital Kinderklinik und Kinderpoliklinik der Ludwig Maximilian Universitat Munchen, Munchen, Bayern, Germany; 6 Partner site Munich, German Centre for Infection Research, Braunschweig, Niedersachsen, Germany; 7 Institute of Infection, Veterinary and Ecological Sciences, University of Liverpool, Liverpool, Merseyside, UK; 8 Alder Hey Children's NHS Foundation Trust, Liverpool, Merseyside, UK; 9 Second Department of Paediatrics, P & A Kyriakou Children's Hospital, National and Kapodistrian University of Athens, Athinon, Greece; 10 Paediatric Immunology, Infectious Diseases & Allergy, Great North Children's Hospital, Newcastle upon Tyne, UK; 11 Newcastle upon Tyne Hospital NHS Trust and Newcastle University, NIHR Newcastle Biomedical Research Centre, Newcastle upon Tyne, Tyne and Wear, UK; 12 Paediatric Infectious Diseases and Immunology, Amalia Children's Hospital, Radboudumc, Nijmegen, Gelderland, The Netherlands; 13 Wilhelmina Children's Hospital, Paediatric Infectious Diseases and Immunology, UMC Utrecht, Utrecht, The Netherlands; 14 Department of General Paediatrics, Medical University of Graz, Graz, Steiermark, Austria; 15 Paediatric Emergency Medicine, Imperial College Healthcare NHS Trust, London, UK; 16 Genetics, Vaccines, Infections and Paediatrics Research group (GENVIP), Hospital Clinico Universitario de Santiago de Compostela, Santiago de Compostela, Galicia, Spain; 17 Department of Infectious Diseases and Faculty of Medicine, Ljubljanski Univerzitetni klinicni center, Ljubljana, Slovenia; 18 Department of Paediatric Infectious Diseases and Immunology, Erasmus MC Sophia Children's Hospital, Rotterdam, Nederland, The Netherlands

**Keywords:** epidemiology, therapeutics

## Abstract

**Objectives:**

To develop and cross-validate a multivariable clinical prediction model to identify invasive bacterial infections (IBI) and to identify patient groups who might benefit from new biomarkers.

**Design:**

Prospective observational study.

**Setting:**

12 emergency departments (EDs) in 8 European countries.

**Patients:**

Febrile children aged 0–18 years.

**Main outcome measures:**

IBI, defined as bacteraemia, meningitis and bone/joint infection. We derived and cross-validated a model for IBI using variables from the Feverkidstool (clinical symptoms, C reactive protein), neurological signs, non-blanching rash and comorbidity. We assessed discrimination (area under the receiver operating curve) and diagnostic performance at different risk thresholds for IBI: sensitivity, specificity, negative and positive likelihood ratios (LRs).

**Results:**

Of 16 268 patients, 135 (0.8%) had an IBI. The discriminative ability of the model was 0.84 (95% CI 0.81 to 0.88) and 0.78 (95% CI 0.74 to 0.82) in pooled cross-validations. The model performed well for the rule-out threshold of 0.1% (sensitivity 0.97 (95% CI 0.93 to 0.99), negative LR 0.1 (95% CI 0.0 to 0.2) and for the rule-in threshold of 2.0% (specificity 0.94 (95% CI 0.94 to 0.95), positive LR 8.4 (95% CI 6.9 to 10.0)). The intermediate thresholds of 0.1%–2.0% performed poorly (ranges: sensitivity 0.59–0.93, negative LR 0.14–0.57, specificity 0.52–0.88, positive LR 1.9–4.8) and comprised 9784 patients (60%).

**Conclusions:**

The rule-out threshold of this model has potential to reduce antibiotic treatment while the rule-in threshold could be used to target treatment in febrile children at the ED. In more than half of patients at intermediate risk, sensitive biomarkers could improve identification of IBI and potentially reduce unnecessary antibiotic prescriptions.

**What is already known on this topic?**

- In children, distinction between invasive bacterial and self-limiting infections on only clinical symptoms is unreliable leading to overuse of antibiotics on the one hand, but to missed invasive bacterial infections in others.
- Several clinical prediction models including biomarkers have been developed to help decision making by risk prediction of patients at high risk or low risk for bacterial infections, but none predicts the outcome invasive bacterial infections in older children or includes children with chronic conditions.

**What this study adds?**

- We derived and externally validated a clinical prediction model based on clinical predictors from the Feverkidstool (clinical symptoms, C reactive protein) and non-blanching rash, neurological symptoms and comorbidity, to early recognise invasive bacterial infections with data from a large observational European-wide study of febrile children aged 0–18 years.
- The rule-out threshold of this model could reduce antibiotic prescription and invasive diagnostics, while the rule-in threshold could be useful to target early treatment for invasive bacterial infections.
- In more than half of the patients at intermediate risk, sensitive new biomarkers could reduce diagnostic uncertainty and improve identification of invasive bacterial infections.

## Introduction

Children presenting at the emergency department (ED) still die from treatable invasive bacterial infections (IBI) due to delayed or missed diagnosis.^1–3^ For not missing one child with IBI, antibiotics are prescribed in children with self-limiting viral infections.^4^ The distinction between bacterial and viral infections based solely on clinical signs and symptoms is unreliable. Although C reactive protein (CRP) and procalcitonin are currently used as markers for bacterial infections, they measure non-specific inflammation and immunological responses. Recent studies focus on proteomic and transcriptomic approaches for finding new discriminators of bacterial and viral infections.^5–8^ Due to costs and limited resources, it is not feasible to apply new biomarkers to all febrile children. Therefore, prediction models are needed to identify risk groups where biomarkers can improve diagnosis.

Clinical prediction models that include clinical signs and CRP or procalcitonin have been developed to assist decision making in treatment of febrile children,^9–15^ and have focused on young infants to differentiate between patients at high risk or low risk for IBI (bacteraemia, meningitis, bone/joint infections). No clinical prediction models for IBI exists for older children who are also at risk for IBI.^16 17^ The Feverkidstool, developed for children aged <16 years, predicts risks for pneumonia and other serious bacterial infections which besides IBIs also includes bacterial infections of the urinary tract, gastrointestinal tract and soft tissue.

Although the Feverkidstool is extensively validated, the original population only included 21 IBI cases and important predictors for IBI such as non-blanching rash or neurological symptoms were not included. Several models yet exist for prediction of bacterial pneumonia and the impact of the original Feverkidstool on antibiotic use in respiratory tract infections is proven.^18^ Therefore, another model for bacterial pneumonia is not required. Furthermore, prediction of urinary tract infections may be less relevant as sensitive laboratory tests (urinalysis) are readily available for accurate diagnosis at ED visit. In addition, the Feverkidstool is developed in previous healthy children and is therefore not applicable for children with chronic conditions with higher risk of IBI. Hence, a new tool is required for early risk assessment of IBI in febrile children including all age ranges (0–18 years) and chronic conditions.

We aim (1) to derive and cross-validate a clinical prediction model including CRP to identify IBIs in febrile children presenting to different European EDs and (2) to identify patient groups which might benefit from new biomarkers.

## Methods

### Study design

This study is embedded in MOFICHE (Management and Outcome of Febrile children in Europe), an observational multicentre study, which is part of PERFORM (PErsonalized Risk assessment in Febrile illness to Optimise Real-life Management across the European Union) (www.perform2020.org).

Children aged from 0 to 18 years with temperature ≥38.0°C or fever <72 hours before ED visit were included. Twelve EDs participated in this study: Austria, Germany, Greece, Latvia, the Netherlands (n=3), Spain, Slovenia and the UK (n=3).^19^ Data were collected for at least 1 year from January 2017 to April 2018. Details of the study design have been described previously.^20^

For this study, we selected patients with CRP measurement and excluded patients with working diagnosis of urinary tract infections after first assessment at the ED.^21^ To identify IBI at the earliest opportunity, we included only the first ED visit for patients with IBI who repeatedly visited the ED within the same disease episode. Data were analysed according to a statistical analysis plan (online supplemental appendix 1).

10.1136/archdischild-2020-319794.supp1 Supplementary data

Collected data included age, sex, comorbidity (chronic condition expected to last ≥1 year),^22^ warning signs for identifying risk of serious illness (National Institute for Health and Care Excellence (NICE))^23^ (consciousness, ill appearance, work of breathing, meningeal signs, focal neurology, non-blanching rash, dehydration) and vital signs (heart rate, respiratory rate, oxygen saturation, temperature, capillary refill time). We collected CRP level (point-of-care or laboratory assay) and microbiologic cultures (blood, cerebrospinal fluid and other) ordered at the ED or at the first day of hospital admission on indication of the physician. Furthermore, we collected data of prescribed antibiotics and admission following ED visit.

### Outcome

IBI included bacterial meningitis, bacteraemia and bacterial bone/joint infections, defined as culture or PCR detection of a single pathogenic bacterium in blood, cerebrospinal or synovial fluid. All cultures that were treated as contaminant and cultures growing contaminants were considered non-IBI (online supplemental appendix 2).^24^ Cultures growing a single contaminant or candida were defined positive in patients with malignancy, immunodeficiency, immunosuppressive drugs or a central catheter, since antimicrobial treatment is needed in these patients.

### Model development

Descriptive and univariate logistic regression analyses were performed for children with and without IBI.

Sample size was estimated based on Riley *et al*.^25^ Assuming 16 predictors, a prevalence of 0.8% and an expected R^2^ of 0.0135 (15% of maximum achievable R^2^), a sample size of 10 587 with 85 cases would be sufficient. For model development,^26 27^ we considered predefined variables with predictive value for IBI: (1) variables in the Feverkidstool^9^ (age, sex, temperature, fever duration, tachypnoea and tachycardia defined by Advanced Paediatric Life Support,^28^ oxygen saturation <94%, capillary refill ≥3 s, work of breathing, ill appearance and CRP value), (2) NICE warnings signs (consciousness, meningeal signs, focal neurology, status epilepticus, non-blanching rash)^23^ and (3) complex chronic condition (≥2 body systems, malignancy or immunocompromised).^22^ Consciousness, meningeal signs and focal neurology were combined into a composite variable abnormal neurology. Linearity of continuous variables was assessed using restricted cubic splines. As in the Feverkidstool, age was modelled linear piecewise for children aged <1 year and >1 year and a logarithmic transformation for CRP was used. Outliers were truncated at the 0.01 percentile for temperature (35.7°C) and the 0.99 percentile for CRP (215 mg/L) and fever duration (8 days).

Variable selection was not influenced by the results of the univariate logistic regression analysis, but was performed using least absolute shrinkage and selection operator (LASSO), which reduces the degree of overfitting by shrinking large regression coefficients (detailed methods in online supplemental appendix 3).^29 30^ The final model was developed on data from all the 12 EDs. For the cross-validation, we created 5 ED groups; 1 group combined the data from the 8 EDs with <10 IBI cases and 4 groups were based on data from EDs with >10 IBI cases per ED: Slovenia, the Netherlands (n=2) and the UK (online supplemental appendix 4). Next, in cross-validation the model was repeatedly derived on four ED groups and validated on the fifth ED group, leading to five different cross-validations.^31^ The five cross-validations were pooled using a random-effects model. This cross-validation determines model performance most accurately and provides information on the heterogeneity of performance across different settings. This cross-validation is therefore superior to a single external validation.^13 31^ We assessed the discriminative ability by the area under the receiver operating curve (AUC), and calibration, the agreement between predicted risks and observed cases. We explored the impact of difference in case-mix heterogeneity on the discriminative ability of the model in the internal-external cross-validation. We used decision curve analysis to evaluate the net benefit of the prediction model.^32^ At different cut-offs for the individual probability of IBI according to the model, we assessed sensitivity, specificity, negative and positive likelihood ratios (LRs). Missing values for the covariates were multiple imputed using the MICE package, resulting in 20 imputation sets (details in online supplemental appendix 3). Sensitivity analysis was performed in the population where missing CRP values were imputed. All analyses were performed in R V.3.6.

## Results

**Figure 1:**
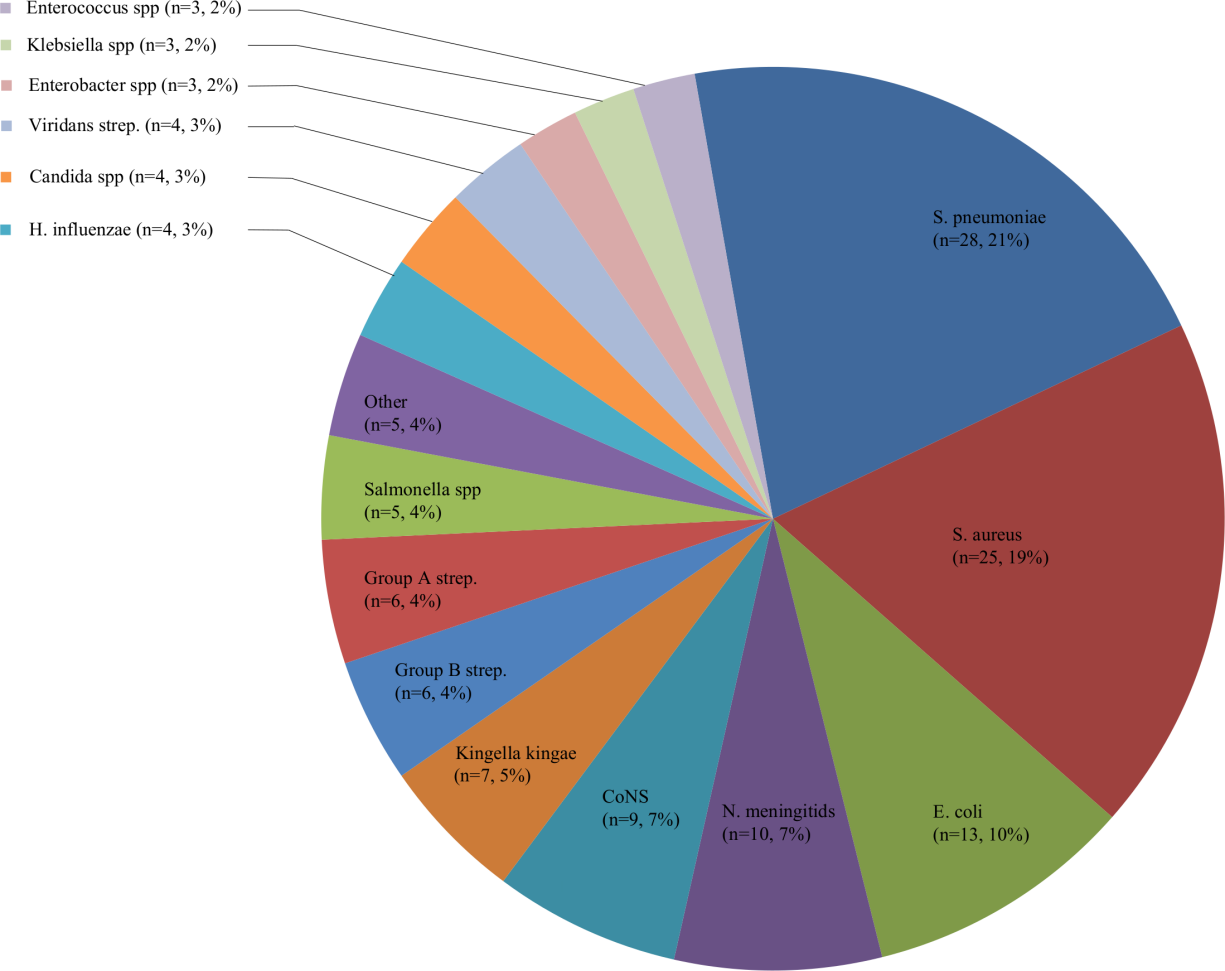
Identified pathogens for invasive bacterial infections (n=135). CoNS, coagulase-negative staphylococci; spp, species.

Of 38 480 patients, 17 213 patients had CRP measurements. Patients with CRP measurements were more often ill-appearing and admitted than patients without CRP measurements (online supplemental appendix 5). We excluded 939 urinary tract infections and 6 repeated visits in the same disease period of patients with IBI, resulting in 16 268 patients. Of those, most common infections were the upper respiratory tract (45%), lower respiratory tract (18%), gastrointestinal tract (14%) and undifferentiated fever (9%). IBI was diagnosed in 135 patients (0.8%), and comprised 119 bacteraemias, 15 bacterial meningitis and 9 bone/joint infections (8 patients had concurrent infections). Main pathogens included *Streptococcus pneumoniae* (21%), *Staphylococcus aureus* (19%), *Escherichia coli* (10%), *Neisseria meningitidis* (7%) and coagulase-negative staphylococcus (7%) (figure 1, online supplemental appendix 6). Complex chronic conditions were present in 37% of patients with IBI vs 6% of patients without IBI. IBI incidence varied from 0.1% to 5.6% of patients per ED (online supplemental appendix 4).

Patients with IBI were similar in age and sex compared with patients without IBI. CRP level was higher in the IBI group (median 62 mg/L, IQR 21–144) than in the non-IBI group (median 16 mg/L, IQR 5–45) (p<0.01) (table 1). The majority of IBIs were treated with antibiotics (n=126, 93.3%) at first ED visit and all were treated with antibiotics in the disease course. The associations of the sole predictors with IBI are provided in online supplemental appendix 7.

**Table 1:** Characteristics of patients with invasive bacterial infections and patients without invasive bacterial infections

	Invasive bacterial infection (n=135)		No invasive bacterial infection (n=16 133)	
	n (%)	Missing	n (%)	Missing
Age in years, median (IQR)	3.2 (0.8–6.0)		2.8 (1.4–6.0)	
Female	76 (56.2)		8932 (55.4)	
Underlying chronic condition		2		89
Any	68 (50.4)		3005 (18.6)	
Complex	50 (37.0)		1008 (6.2)	
Referred	96 (71.1)	3	8633 (53.5)	936
Triage urgency		5		477
Low: standard, non-urgent	41 (30.4)		9242 (57.3)	
High: immediate, very urgent, intermediate	89 (65.9)		6414 (39.8)	
Feverkidstool				
Temperature in °C, median (IQR)	38.0 (37.4–38.7)	3	37.8 (37.0–38.5)	764
Fever duration in days, median (IQR)	0.5 (0.5–3)	5	1.5 (0.5–3)	817
Tachypnoea (APLS)	38 (28.1)	37	3345 (20.7)	3919
Tachycardia (APLS)	81 (60.0)	5	5578 (34.6)	821
Hypoxia <95%	4 (2.9)	13	749 (4.6)	2373
Prolonged capillary refill (>3 s)	8 (5.9)	29	305 (1.9)	2311
Increased work of breathing	11 (8.1)	40	887 (5.5)	2136
Ill appearance	60 (44.4)	13	4398 (27.3)	610
CRP in mg/L, median (IQR)	61 (21–144)		16 (5–45)	
NICE warning signs				
Decreased level of consciousness	6 (4.4)		137 (0.8)	141
Meningeal signs	8 (5.9)	24	116 (0.7)	845
Focal neurology	2 (1.5)	29	95 (0.6)	1249
Status epilepticus	0 (0.0)	8	49 (0.3)	887
Rash: petechiae/non-blanching	10 (7.4)	25	640 (3.9)	1183
Blood cultures performed	134 (99.3)		3002 (18.6)	
CSF performed	25 (18.5)		381 (2.4)	
Admission to the ward >24 hours	111 (82.2)	1	5879 (36.4)	159
Admission to the ICU	10 (7.4)		125 (0.8)	17
Antibiotic treatment following ED visit	126 (93.3)		5804 (35.9)	197
LSI: airway, breathing or haemodynamic support	16 (11.9)		343 (2.1)	

APLS, advanced paediatric life support; CRP, C reactive protein; CSF, cerebrospinal fluid; ED, emergency department; ICU, intensive care unit; LSI, life-saving intervention; NICE, National Institute for Health and Care Excellence.

**Figure 2:**
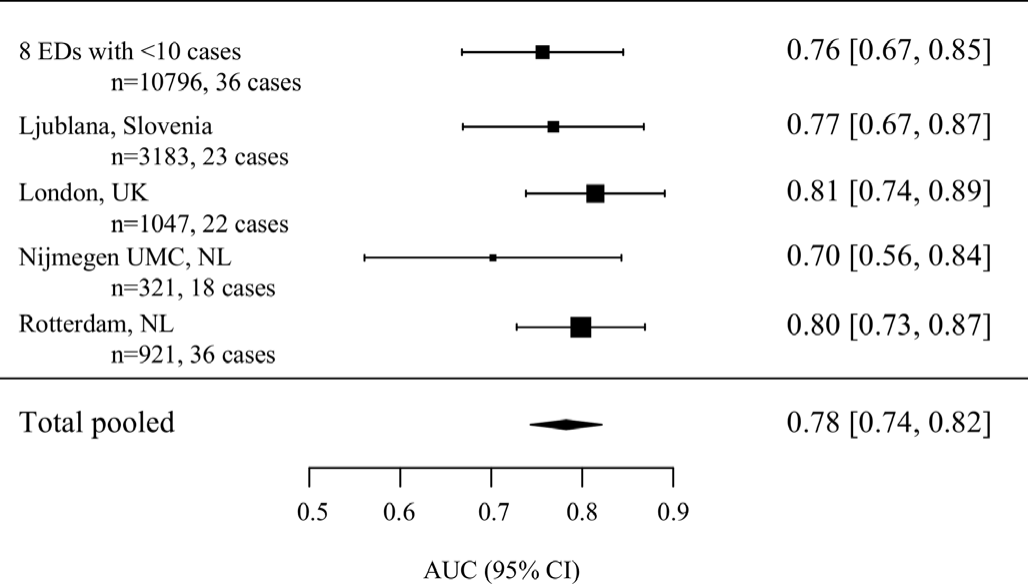
Discriminative value of the prediction model for invasive bacterial infection for five internal-external cross-validations. The model was repeatedly derived on four ED groups, and validated on the fifth ED group which was left out from the derivation. The five cross-validations were pooled using a random-effects model. More details are provided in figure A in online supplemental appendix 3. AUC, area under the receiver operating curve; ED, emergency department; NL, The Netherlands; UK, United Kingdom; UMC, University Medical Centre.

The final model is presented in table 2. This model discriminated well (AUC 0.84 (95% CI 0.81 to 0.88)). In the cross-validation, the model discriminated moderate to well (range AUC 0.76–0.81) yielding a pooled AUC of 0.78 (95% CI 0.74 to 0.82) (figure 2). Calibration was poor to moderate for the different cross-validations (range slope: 0.45–0.81, range intercept −1.2 to 1.0) (online supplemental appendix 8). Apparent calibration was improved by adding an ED-specific variable for high (>2%) versus low (<2%) incidence of IBI (online supplemental appendix 9).

**Table 2:** Model specification of multivariate logistic model for IBI

		Coefficients	OR
	(Intercept)	−9.16	0.00
Feverkidstool	Male	−0.19	0.83
	Age <1 year*	−2.53	0.08
	Age ≥1 year*	0.00	1.00
	Temperature	−0.05	0.95
	Fever duration in days	−0.15	0.86
	Tachypnoea	−0.44	0.65
	Tachycardia	0.69	2.00
	Hypoxia	−0.87	0.42
	Increased work of breathing	−0.31	0.73
	Ill appearance	0.87	2.38
	ln CRP	0.76	2.14
NICE warning signs	Abnormal neurology	1.54	4.66
	Non-blanching rash	1.38	3.96
Comorbidity	Complex chronic condition	2.41	11.1

The risk of children aged <1 year was calculated: β_(age <1 year)_×age in years.

The risk of children aged ≥1 year was calculated: β_(age <1 year)_×1+(age in years−1)×β (_age ≥ 1 in years_).

*Age <1 year and age ≥1 year were calculated linear-piecewise.

CRP, C reactive protein; IBI, invasive bacterial infection; ln, natural log.

The diagnostic performance was good for the rule-out threshold of 0.1% with sensitivity of 0.97 (95% CI 0.93 to 0.99) and negative LR of 0.09 (95% CI 0.03 to 0.23) (table 3, online supplemental appendix 10). For the rule-in threshold of 2.0%, the model had specificity 0.94 (95% CI 0.94 to 0.95) and positive LR of 8.4 (95% CI 6.9 to 10.0). The intermediate thresholds of 0.1%–2.0% performed poorly (ranges: sensitivity 0.59–0.93, negative LR 0.14–0.47, specificity 0.52–0.88, positive LRs 1.9–4.8) and comprised 9784 (60.1%) patients. The rule-in threshold misclassified four patients with IBI from three different EDs, including two patients with arthritis, and two patients with a sinusitis and pneumonia resulting in bacteraemia. Three of these patients had CRP levels <10 mg/L and symptoms <1 day.

**Table 3:** Diagnostic performance of the prediction model for different risk thresholds for invasive bacterial infection

Risk thresholds (%)	N below threshold (%)	N above threshold (%)	Sensitivity (95% CI)	Negative LR (95% CI)	Specificity (95% CI)	Positive LR (95% CI)
**0.1**	5495 (33.8)	10 773 (66.2)	0.97 (0.93 to 0.99)	0.09 (0.03 to 0.23)	0.34 (0.33 to 0.35)	1.5 (1.4 to 1.5)
**0.2**	8461 (52.0)	7807 (48.0)	0.93 (0.87 to 0.96)	0.14 (0.08 to 0.26)	0.52 (0.52 to 0.53)	1.9 (1.9 to 2.1)
**0.25**	9416 (57.9)	6852 (42.1)	0.90 (0.84 to 0.95)	0.17 (0.10 to 0.28)	0.58 (0.58 to 0.59)	2.2 (2.0 to 2.3)
**0.5**	12 200 (75.0)	4068 (25.0)	0.76 (0.67 to 0.83)	0.32 (0.24 to 0.44)	0.75 (0.75 to 0.76)	3.1 (2.8 to 3.4)
**1.0**	14 224 (87.4)	2044 (12.6)	0.59 (0.50 to 0.67)	0.47 (0.39 to 0.58)	0.88 (0.87 to 0.88)	4.8 (4.1 to 5.6)
**2.0**	15 279 (93.9)	989 (6.1)	0.48 (0.39 to 0.57)	0.55 (0.47 to 0.65)	0.94 (0.94 to 0.95)	8.4 (6.9 to 10)
**5**	15 831 (97.3)	437 (2.7)	0.36 (0.37 to 0.45)	0.65 (0.57 to 0.74)	0.98 (0.97 to 0.98)	15 (12 to 19)

LR, likelihood ratio.

In sensitivity analysis involving the population with imputed CRP levels (n=37 093, IBI n=135), model development yielded similar coefficients (online supplemental appendix 11).

## Discussion

Based on the Feverkidstool and important predictors for early recognition of IBI, we derived and cross-validated a clinical prediction tool, in febrile children at different European EDs. The prediction model discriminated well between patients with and without IBI. The risk threshold of 0.1% has good rule-out value for IBI and thus decreases the risk of missing an IBI. The higher risk thresholds of >2.0% have good rule-in value and these thresholds can be used to identify patients at high risk of IBI to target treatment. The large number of patients with intermediate risk of 0.1%–2.0% for IBI is expected to benefit most from sensitive biomarkers.

Strengths of this study include the participation of 12 European EDs based in 8 countries with a broad population of febrile children of all ages and chronic conditions. Furthermore, we performed five cross-validations which provided us insight in heterogeneity between EDs, and improves the generalisability of our results. Second, we included a large number of IBI cases, while previous studies did not have sufficient cases to define a prediction model exclusively for IBI.^9–11^ Furthermore, our model involves accessible predictors as clinical symptoms and CRP level, which will facilitate implementation in practice. We provide clinical case examples of the model (online supplemental appendix 12) and, to help physicians to use this model in practice, a web-based digital calculator will be developed.

Our study has some limitations. First, we focused our study on patients who had CRP measurement on indication. This involved more severe illness than patients without CRP measurement. However, the CRP group reflect patients with diagnostic uncertainty and is more likely to benefit from a clinical prediction model. All patients with IBI had CRP measurement, leading to inclusion of all eligible IBIs in the main analysis. In our sensitivity analysis, predictors were similar in the model developed on imputed CRP levels. Therefore, model performance was not influenced by selection of patients with CRP measurement. Second, diagnostic tests were ordered according to usual care. If patients with an IBI did not have cultures taken >24 hours after hospital admission, this was not included in the data and these patients could have been misclassified as non-IBI. Since diagnostic workup is in general performed at the ED or <24 hours after presentation, this misclassification is minimised. Third, due to the low incidence of IBI, model performance was evaluated in cross-validation with a lower number of cases than is optimal for validation (100 cases).^33 34^Although discrimination of the model was good in the cross-validations, calibration was poor to moderate. The low incidence of IBI and other case-mix differences not taken into account by our model may have influenced model performance in the cross-validation. Our range of IBI incidence (range EDs 0.1%–5.6%) was comparable with IBI incidence in other studies including febrile population of all age ranges (range 0.4%–4.5%).^9 11 35^ Fourth, due to limited measurements of systolic blood pressure (14.7%) and procalcitonin in our cohort (1.6%), we were not able to include these as predictor. Lastly, data on individual immunisation status were not available and were not included in the model. In the clinical assessment of febrile patients, immunisation status should be taken into account.

Patients with and without IBI were discriminated well in the cross-validations. Calibration was poor to moderate indicating discrepancy between model predictions and the observed risk of IBI. Addition of the ED covariate of low/high incident IBI improved calibration, indicating that model performance is influenced by the likelihood of IBI in the ED. Therefore, ED incidence should be included in the model.

Clinical prediction models involving older children are the Feverkidstool and Irwin’s model, and predict pneumonia and other serious bacterial infections separately, whereas our model focuses on IBI. Discrimination of our model in cross-validation (pooled AUC: 0.78 (95% CI 0.74 to 0.82) was better compared with one external validation and similar to another external validation of the Feverkidstool for other serious bacterial infection.^9 11^ Unlike our study, these models were not based on an European-wide ED population. We recommend to use the Feverkidstool to guide antibiotic prescription in suspected lower respiratory tract infections^18^ and to use our model in febrile children to predict IBI. These two models, the original Feverkidstool and our model will be integrated in one electronic decision tool. For both implementation of the Feverkidstool and our model, measurement of (point-of-care) CRP is necessary. We do not recommend CRP measurement in all febrile children, but since CRP level is an important discriminator in bacterial and viral illness, measurement should be easily accessible to aid in the decision-making process at the ED.

Missing and undertreatment of IBI in children can lead to morbidity and mortality. Current practice is to start antibiotic treatment in patients at risk for bacterial infection awaiting culture results which take >48 hours. Since the low incidence of IBI, this leads to overuse of antibiotics and resources. The balance of not missing IBIs and overtreating self-limiting infections is delicate. Therefore, clinical prediction models can help in decision making at the ED. Our study showed that the low-risk threshold can be helpful to rule-out IBI and to reduce invasive diagnostics and antibiotic use.

Starting early treatment is key to prevent adverse outcomes due to IBI. The high risk threshold of >2.0% can be used for targeted treatment with intravenous antibiotics. Although our model was able to identify 38% of the study population as low or high risk, diagnostic uncertainty exist for the intermediate group (60%). In our study, this intermediate group with diagnostic uncertainty was estimated as 25% of the population of febrile children presenting to the ED, including patients without CRP measurement. Additional diagnostics including procalcitonin, repeated CRP measurement^36^ or novel sensitive biomarkers may be helpful in the decision making for this intermediate risk group. The potential benefit of additional diagnostics using these risk thresholds will need to be evaluated in future studies.

## Conclusion

Based on the Feverkidstool and important clinical predictors, we derived and cross-validated a clinical prediction model for early detection of IBI in febrile children in an European-wide cohort. Where the rule-in threshold of this model could target early treatment to reduce adverse outcomes from IBI, the rule-out threshold has the potential to reduce unnecessary use of invasive diagnostics and antibiotics. However, more than half of the population was at intermediate risk. In this group, sensitive, new biomarkers could improve identification of IBI and could potentially reduce unnecessary antibiotic use.

## Data Availability

An anonymized data set containing individual participant data is available in a public data repository: https://data.hpc.imperial.ac.uk/resolve/?doi=7549. DOI: 10.14469/hpc/7549. For inquiries to obtain the full dataset, please contact the data manager of the PERFORM consortium (Tisham.de08@imperial.ac. uk).
