# Supplementary material for: Development and validation of a prediction model for invasive bacterial infections in febrile children at European Emergency Departments: MOFICHE, a prospective observational study
Source: Arch Dis Child. 2020 Nov 18;106(7):641–7. doi: 10.1136/archdischild-2020-319794 (PMC8237171; doi:10.1136/archdischild-2020-319794)
Supplement: Supplementary data [file archdischild-2020-319794supp001.pdf]

Supplemental information

Appendix 1: Statistical analysis plan

Statistical Analysis Plan

Prediction of invasive bacterial infections in febrile children presenting to Emergency Departments in Europe

SAP version 1.0 date 14th July 2019

Background

Still today children die on treatable infectious diseases due to delayed or missed diagnosis presented at the Emergency Department (ED) or primary care.(1-3) On the other hand, antibiotics are prescribed for viral infections and infection with an unknown bacterial or viral cause in order not to miss one child with an invasive bacterial infection.(4)

The distinction between invasive bacterial infections and viral infections on only clinical signs and symptoms is difficult. Biomarkers as C-reactive protein and procalcitonin are currently used in febrile children to detect bacterial infections and to target appropriate antibiotic prescribing. However, these markers measure non-specific inflammation and immunologic responses. Recent research focuses on finding new discriminators of bacterial and viral infections using novel, sophisticated techniques (genomic, proteomic and transcriptomic approaches).(5-7) It is yet unclear which patients would benefit from potential new biomarkers. It is not feasible to apply new biomarkers to all febrile children. Therefore, decision models need to be developed which can identify these patients.

We searched PUBMED from 1st January 2009 to 1st July 2019 for published studies covering clinical prediction models for bacterial infections in children using keywords "child", "fever", "bacterial infection" and "clinical prediction" and checked references for relevant articles. The existing literature on clinical prediction models for bacterial infections focuses on young infants (< 3 months) and healthy children in particular. For older children, the Feverkidstool (Nijman et al.) is an extensively validated clinical prediction model for prediction of pneumonia and other serious bacterial infections which includes bacteraemia and meningitis but also infections of the urinary tract, gastro-intestinal tract and soft tissue. We could not identify a clinical prediction model for the outcome invasive bacterial infections including older children or children with chronic conditions.

Objectives

1. To update an existing clinical prediction model to identify invasive bacterial infections in febrile children at the ED
2. Can we target patients who can benefit from a new biomarker based on risk-prediction by this model?

Methods

Study design:
Prospective observational study
This study is a prospectively planned analysis in the MOFICHE study (Management and Outcome of Febrile Illness in Children) which is part of the PERFORM project. MOFICHE is a prospective observational study using routine data. The need for informed consent was waived.

Setting:
12 Emergency Departments (EDs) in 8 countries

Population:
Children 0-18 years with fever (temperature >38.0 C) measured at ED or history of fever (<72 hours) before ED visit. For this analysis, we will exclude children with working diagnosis of urinary tract infections after ED visit. For diagnosis of urinary tract infections, easy available diagnostics are already available at the ED. Therefore, a clinical prediction model has limited additional value in this group. Furthermore, we will focus our analysis on patients with CRP measurement since these are patients with diagnostic uncertainty after initial assessment by the physician.

Inclusion period:

1 January 2017 – 1 April 2018, at least 12 months per study site.

Primary outcomes:
Invasive bacterial infections (IBI): bacteraemia, bacterial meningitis and bacterial bone and join infections. Infections were defined positive growth of a single pathogenic bacterium in blood, cerebrospinal fluid or synovial fluid from cultures collected at ED visit or the first 24 hours from hospital admission.

Cultures growing contaminants (coagulase-negative staphylococci, alpha-haemolytic streptococci, *Micrococcus* species or *Propionibacterium* species are defined negative (8)

In children who are immunocompromised, malignancies or with a central line, these contaminants are still relevant invasive bacterial infections that need antibiotic treatment. In these patient groups, cultures with a single contaminant are defined positive.

All patients were entered in the electronic case record form (eCRF) by the local team. We will check all the positive cultures to ensure consistency and validity of coding.

Missing data
For this analysis, we will exclude patients with no CRP value and exclude patients with working diagnosis of urinary tract infection. We will use multiple imputation by chained equations using the MICE package in R to impute all missing predictor variables. We will assume the variables to be ‘missing at random’ where missingness can be explained by other variables in the data. We will incorporate hospital, all predictor variables, outcome measures and other auxiliary variables in the imputation model.
Multiple imputation will be performed on all patients (n=38480).

Variables in the multiple imputation model:

**General characteristics**	**Markers of disease severity**	**Vital signs**	**Diagnostics**	**Treatment**	**Outcomes**
Hospital	Triage urgency	Heart rate	CRP-level	Immediate life-saving interventions	Disposition
Age	Fever duration	Respiratory rate	Chest X-ray categories	Oxygen treatment	Final diagnosis
Sex	Capillary refill time	Temperature	Urinalysis categories	Inhalation medication	Focus of infection
Referral type (self / GP / emergency services / other)	Ill appearance	Oxygen saturation	Blood culture performed	Antibiotic prescription type	
Previous medical care (yes, primary care / yes, this ED / yes other secondary care)	Work of breathing		Cerebrospinal fluid performed	Antibiotic prescription mode	
Season	Meningeal signs			Previous antibiotic treatment	
Arrival hours (morning / evening / night)	Focal neurology				
Comorbidity	Non-blanching rash				

	Dehydration				
	Seizures				

Descriptive analysis
We will perform descriptive analysis for children with and without IBI. We will use frequencies, mean and standard deviation for normally distributed data, median and interquartile range for normally distributed data. In addition, we will compare patients with CRP measurement and patients without CRP measurement.

Predictor variables
We will include predictor variables chosen a-priori that have predictive value for bacterial infection. We will perform univariate logistic regression analysis for these predictor variables:

Predictor variables included in the Feverkidstool (9):
- Age
- Sex
- Temperature
- Fever duration in days
- Tachypnea: defined by Advanced Paediatric Life Support (10)
- Tachycardia: defined by Advanced Paediatric Life Support (10)
- Hypoxia: oxygen saturation <94%
- Prolonged capillary refill time: >3 seconds
- Increased work of breathing: chest wall retractions, nasal flaring, grunting or apnoea
- Ill appearance: ill, moderately ill, irritable or uncomfortable
- C-reactive protein value

NICE red warning signs for serious illness (11):
- Abnormal consciousness: responsive to verbal stimulation, responsive to pain or unresponsive
- Presence of meningeal signs: presence of Kernig, Brudzinski, tripod phenomenon, neck stiffness or bulging fontanelle
- Focal neurological signs
- Status epilepticus: seizures for >=30 minutes
- Non-blanching rash: petechiae or other non-blanching rash

Complex chronic condition (12)
- Chronic condition in ≥2 body systems that is expected to last at least 1 year or malignancy or immunocompromised

We will use 10 events per variable to include predictor variables in model development. If not enough events are available, we will combine abnormal consciousness, presence of meningeal signs and focal neurological signs in a composite variable.
Linearity of continuous variables will be assessed using restricted cubic splines. Outliers for continuous variables will be truncated at the 0.01 percentile and the 0.99 centile.

Model development
We will perform variable selection by least absolute shrinkage and selection operator (LASSO). Using LASSO, we perform variable selection and reduce degree of overfitting by shrinking large regression coefficients.(13) We will estimate the lambda using 10 times 10-fold-cross validation. To note, variable selection will not be based on significance in univariate logistic regression analysis.

Model validation
The model will be validated using internal-external cross-validation. In this method, the model is repeatedly derived on all EDs except one, and validated on the remaining ED.(14, 15)

Model performance
Model performance will be assessed by

- Discrimination of the model by concordance (c)-statistic.
- Calibration, the agreement between predicted risks and observed outcome will be visualized using calibration plots.(16)
- Diagnostic performance at different risk-threshold for the probability of IBI using sensitivity, specificity and negative and positive likelihood ratios. We will focus on cut-offs that can be used to rule-out (negative LR <0.2) or rule-in IBI (positive LR>5).(17)

Sensitivity analysis
A sensitivity analysis will be performed in the population where missing CRP values will be imputed.

Drafted by: Nienke N. Hagedoorn
Statistician: Daan Nieboer
Supervision: Dr. Clementien Vermont, Prof. Henriette A. Moll

Appendix 2: Definition of contaminants

**Appendix 3: Definition of contaminants**
Micrococcus
Coagulase-negative staphylococci
Propionibacterium species
Alpha-haemolytic streptococci (except pneumococcus)
Corynebacterium species (diphteroids)
Bacillus species
Pseudomonas (except P. aeruginosa)
Other environmental non-fermenting gram-negative rods

Appendix 3: Additional methods on data analysis

Multiple imputation

Missing data were multiple imputed using the MICE package in R v3.4. The imputation model included the outcome variable IBI, all considered predictors, ED and other auxiliary variables related to casemix and disease severity (specific details of the multiple imputation model are proved in the Statistical Analysis Plan). The imputation process resulted in 20 imputation sets. For all the statistical analysis, apart from the model development in LASSO (least absolute shrinkage and selection operator), results were pooled for a final result.(18) The LASSO was applied to a stacked dataset containing all imputed data.(19) To adjust for the inflated sample size we assigned each record a weight of 1/20 (20 is number of imputed datasets).

Model development and internal-external cross-validation

For model development (20, 21), we considered predefined variables with predictive value for IBI: 1) variables in the Feverkidstool(9) (age, sex, temperature, fever duration, tachypnea and tachycardia defined by Advanced Pediatric Life Support(10), oxygen saturation <94%, capillary refill >=3 seconds, work of breathing, ill appearance and CRP value), 2) NICE warnings signs which were not included in the Feverkidstool (consciousness, meningeal signs, focal neurology, status epilepticus, non-blanching rash)(11) and 3) complex chronic condition (condition in ≥2 body systems, malignancy or immunocompromised).(12) Level of consciousness, meningeal signs and focal neurology were combined into a composite variable abnormal neurology. Linearity of continuous variables was assessed using restricted cubic splines. As in the Feverkidstool, age was modelled linear piecewise for children <1 year and children >1 year and a logarithmic transformation for CRP was used. Outliers were truncated at the 0.01 percentile for temperature (35.7 °Celsius) and the 0.99 percentile for CRP (215 mg/L) and fever duration (8 days).

Variable selection was not influenced by the results of the univariate logistic regression analysis, but was performed using least absolute shrinkage and selection operator (LASSO).(13, 22) This approach aims to reduce the degree of overfitting by shrinking large regression coefficients and performs variable selection.(13) The lambda to derive the final model was estimated using 10 times 10-fold cross-validation. We used internal-external cross-validation in EDs with >10 IBI cases (four EDs) and EDs with <10 IBI cases (eight EDs) were combined in one group leading to five ED groups (appendix 5). In internal-external cross-validation The model was repeatedly derived on all ED groups except one, and validated on the remaining ED group (see figure A below).(14) Unlike splitting data in a derivation and validation set, this method uses all available data for the model development and uses cross-validation to validate the model five times. This cross-validation determines model performance most accurately but also provides information on the heterogeneity of performance across different settings. This internal-external cross-validation is therefore superior to a single external validation.(14, 15) We assessed the discriminative ability by the area under the receiver operating curve (AUC), and calibration, the agreement between predicted risks and observed cases., was evaluated by calibration plots. We explored the impact of difference in case-mix heterogeneity on the discriminative ability of the model in the internal-external cross-validation. Sensitivity, specificity, negative and positive likelihood ratios (LR) were evaluated at different cut-offs for the individual probability of IBI according to the model. We explored cut-offs for ruling-out (negative LR <0.2) or ruling-in IBI (positive LR >5).(17) Missing values for the covariates were multiple imputed (MICE). Sensitivity analysis was performed in the population where missing CRP values were imputed. All analyses were performed in R v3.6.

Figure A

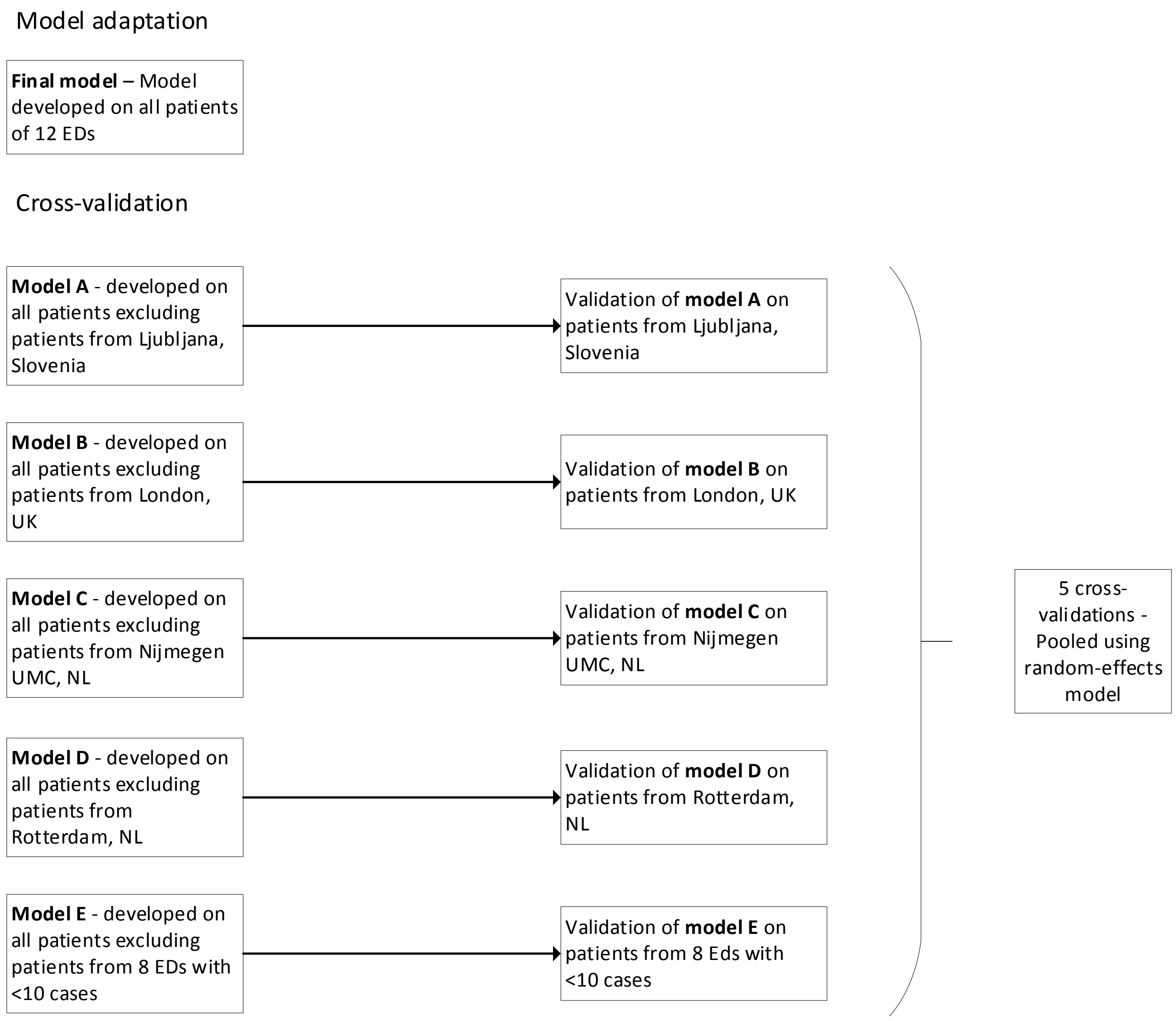
Model adaptation
Final model – Model developed on all patients of 12 EDs
Cross-validation
Model A - developed on all patients excluding patients from Ljubljana, Slovenia
Validation of model A on patients from Ljubljana, Slovenia
Model B - developed on all patients excluding patients from London, UK
Validation of model B on patients from London, UK
Model C - developed on all patients excluding patients from Nijmegen UMC, NL
Validation of model C on patients from Nijmegen UMC, NL
Model D - developed on all patients excluding patients from Rotterdam, NL
Validation of model D on patients from Rotterdam, NL
Model E - developed on all patients excluding patients from 8 EDs with <10 cases
Validation of model E on patients from 8 Eds with <10 cases
5 cross-validations - Pooled using random-effects model

Appendix 4: EDs - classification of EDs with low (<2%) and high incidence (>2%) for IBI based on proportion of invasive bacterial infection, and proportion of chronic complex comorbidity per ED

ED	N total included patients	N study population	IBIs N (% of study population per ED)	Chronic complex comorbidity N (% of study population per ED)
Graz, Austria	2241	1987	1 (0.1%)	73 (3.7%)
Athens, Greece	4548	1450	1 (0.1%)	19 (1.3%)
Riga, Latvia	9000	5495	9 (0.2%)	60 (1.1%)
Munich, Germany	1173	456	1 (0.2%)	19 (4.2%)
Nijmegen, CWZ, the Netherlands	423	184	1 (0.5%)	12 (6.5%)
Ljubljana, Slovenia	3667	3183	23 (0.7%)	61 (1.9%)
Liverpool, UK	1623	468	8 (1.7%)	76 (16.2%)
Newcastle, UK	3854	475	9 (1.9%)	41 (8.6%)
London, UK	5714	1047	22 (2.1%)	184 (17.6%)
Santiago de Compostela, Spain	3877	281	6 (2.1%)	9 (3.2%)
Rotterdam, the Netherlands	1683	921	36 (3.9%)	369 (40.1%)
Nijmegen, UMC, the Netherlands	677	321	18 (5.6%)	135 (42.1%)
**Total**	**38480**	**16268**	**135**	**1058**
EDs with low incidence for IBI (<2%)		13698	53 (0.4%)	367 (2.7%)
EDs with high incidence for IBI (>2%)		2570	82 (3.2%)	364 (14.2%)

ED, emergency department; IBI, invasive bacterial infection; UK, United Kingdom; UMC, university medical centre; CWZ, Canisius Wilhelmina Hospital

Appendix 5: Patient characteristics of patients with CRP measurement and patients without CRP measurement

	CRP measured (n=17,213)			No CRP measured (n=21267)		
	n (%)	Range EDs	Missing	n (%)	Range EDs	Missing
**General characteristics**						
**Age in years,** median (IQR)	2.77 (1.29-6.02)			2.74 (1.31-5.28)		
**Male**	9305 (54.1)	49.6-62.0		11805 (55.5)	52.4-62.4	1
**Previous chronic condition**			97			273
Any	3332 (19.4)	7.8-71.8		3162 (14.9)	3.9-61.6	
Complex	1138 (6.6)	1.1-41.3		729 (3.4)	0.0-32.6	
**Referred**	9287 (53.9)	6.9-99.2	980	6789 (31.9)	3.9-99.3	185
**Triage urgency**			529			647
Low: standard, non-urgent	9794 (56.9)	10.9-86.5		14291 (67.2)	8.8-93.9	
High: immediate, very urgent, intermediate	6890 (40.0)	13.5-86.8		6329 (29.8)	6.1-89.9	
**Feverkidstool**						
Temperature in °C, median (IQR)	37.8 (37-38.5)		809	37.7 (36.9-38.4)		2211
Fever duration in days, median (IQR)	1.5 (0.5-3)		875	1.5 (0.5-3.0)		1900
Tachypnea (APLS)	3585 (20.8)	5.9-45.8	4186	4942 (23.2)	2.4-48.3	4607
Tachycardia (APLS)	6001 (34.9)	11.0-54.9	887	6854 (32.2)	11.4-49.4	2620
Hypoxia <95%	762 (4.4)	1.3-9.2	2538	733 (3.4)	0.3-12.9	3043
Prolonged capillary refill (>3 sec)	339 (1.9)	0.2-7.0	2503	84 (0.4)	0.0-2.6	1928
Work of breathing	913 (5.3)	0.5-13.2	2315	1732 (8.1)	0.0-35.6	3176
Ill appearance	4742 (27.5)	1.9-52.6	664	1265 (5.9)	0.4-43.3	1057
CRP in mg/L, median (IQR)	17 (5-49)		7	NA		
**NICE Warning signs**						
Decreased consciousness	148 (0.9)	0.1-5.4	150	53 (0.2)	0.0-1.8	240
Meningeal signs	126 (0.7)	0.1-3.7	943	11 (0.1)	0.0-0.1	1101
Focal neurology	102 (0.6)	0.0-3.7	1376	31 (0.1)	0.0-1.8	1081
Status epilepticus	51 (0.3)	0.0-2.3	940	15 (0.1)	0.0-1.2	201
Rash: petechiae/non blanching	664 (3.9)	1.1-18.0	1307	448 (2.1)	0.4-4.1	3106
**Blood cultures performed**	3478 (20.2)	0.5-73.7		88 (0.4)	0.0-2.0	
**CSF performed**	444 (2.6)	0.2-13.5		8 (0.0)	0.0-0.2	
**Admission to the ward >24 hours**	6590 (38.3)	18.0-63.8	175	668 (3.1)	0.9-29.6	347
**Admission to the ICU**	135 (0.8)	0.3-5.4	21	23 (0.1)	0.0-2.4	35
**Antibiotic treatment following ED visit**	6795 (39.5)	27.9-70.8	211	5504 (25.9)	16.9-43.0	273
**Lifesaving interventions:** airway, breathing or hemodynamic support	371 (2.2)	0.0-11.5		112 (0.5)	0.0-3.0	
**Urinary tract infection**	935 (5.4)	3.2-9.7	4	418 (1.9)	0.9-3.8	23

APLS, advanced paediatric life support; CRP, C-reactive protein; CSF, cerebrospinal fluid; ED, emergency department; ICU, intensive care unit; IQR, interquartile range; NA, not applicable

Appendix 6: Details of patients with complex chronic conditions

Identified pathogen stratified for complex chronic comorbidity

Identified pathogen	No complex chronic condition , n=85<br>n (%)	Complex chronic condition , n=50<br>n (%)
Strep. pneumoniae	23 (27.1%)	5 (10%)
Staph. aureus	15 (17.6%)	10 (20%)
E. coli	9 (10.6%)	4 (8%)
Neisseria meningitidis	9 (10.6%)	1 (2%)
Kingella kingae	7 (8.2%)	0 (0%)
Group B streptococcus	6 (7.1%)	0 (0%)
Group A streptococcus	5 (5.9%)	1 (2%)
Salmonella spp	4 (4.7%)	1 (2%)
Haemophilus influenzae	4 (4.7%)	0 (0%)
Enterobacter spp	2 (2.4%)	1 (2%)
Coagulase-negative staphylococci (CoNS)		9 (18%)
Candida species		4 (8%)
Viridans streptococci		4 (8%)
Klebsiella spp		3 (6%)
Enterococcus spp		3 (6%)
Moraxella spp		1 (2%)
Other	1 (1.2%)	3 (6%)

C-reactive protein level in immunocompromised patients for no IBI (A) vs IBI (B) for IBI risk categories

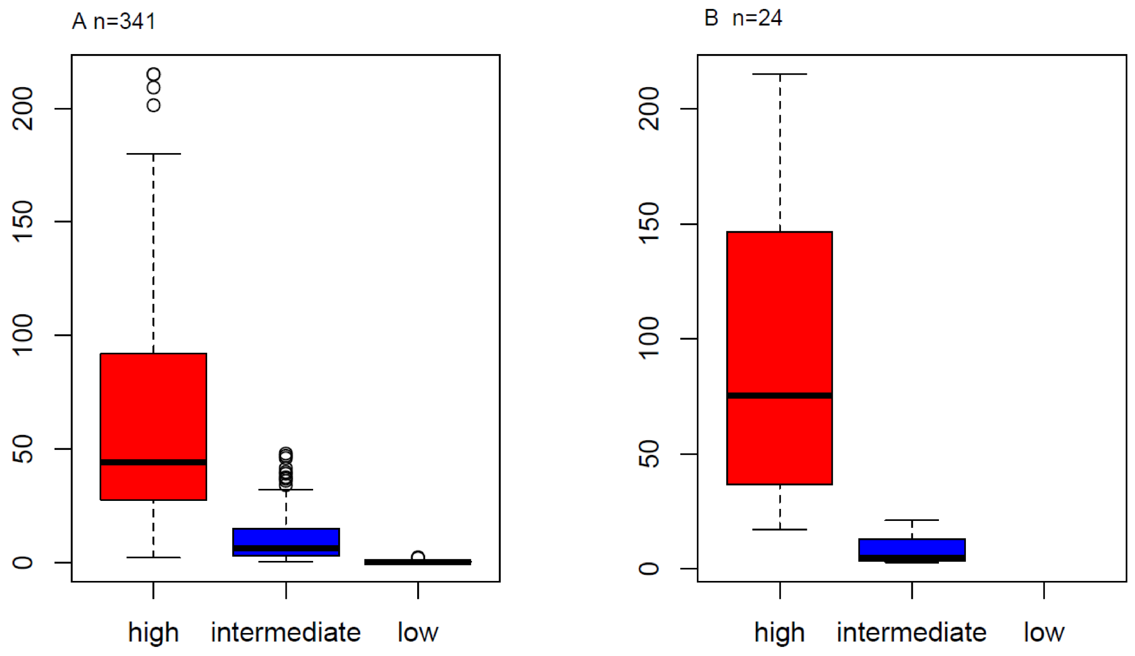

Appendix 7: Univariate logistic regression analysis for invasive bacterial infection.

Supplementary file 5:
Univariate logistic regression analysis for invasive bacterial infection.
N=16268, IBI cases N=135

Variables	OR (95%CI)*
**Feverkidstool**	
Male	1.04 (0.74-1.46)
Age <1 year±	0.25 (0.14-0.43)*
Age >1 year±	1.01 (0.97-1.05)
Temperature in °C	1.34 (1.13-1.59)*
Fever duration in days	0.89 (0.80-0.99)*
Tachypnea (APLS)	1.50 (1.03-2.18)*
Tachycardia (APLS)	2.84 (2.01-4.01)*
o2 saturation <94%	0.65 (0.24-1.75)
Prolonged capillary refill time (>3 sec)	2.62 (1.24-5.56)*
Presence of work of breathing	1.62 (0.90-2.93)
Ill appearance	2.51 (1.76-3.58)*
Ln CRP	1.89 (1.63-2.19)*
**NICE alarming signs**	
Status epilepticus	No cases
Reduced level of consciousness	4.70 (2.04-10.83)*
Focal neurology	2.30 (0.54-9.71)
Meningeal signs	9.20 (4.54-18.62)*
Abnormal neurology: decreased level of consciousness, presence of meningeal signs or focal neurology	4.81 (2.61-8.91)
Non-blanching rash	2.31 (1.21-4.41)*
**Chronic condition**	
Complex chronic condition	8.83 (6.19-12.59)*

*Significant, $p<0.05$

±The risk of children aged < 1 year was calculated: $\beta_{(age < 1\ year)} \times$age in years.
The risk of children aged >1 years was calculated with: $\beta_{(age < 1\ year)} \times 1 + \beta_{(age \geq 1\ year)} \times$(age in years−1).

APLS, Advanced Paediatric Life Support; CRP, C-reactive protein; ln, natural log

Appendix 8: **Calibration plot: observed proportion vs predicted probability of the clinical prediction model for 5 internal-external cross-validations.**

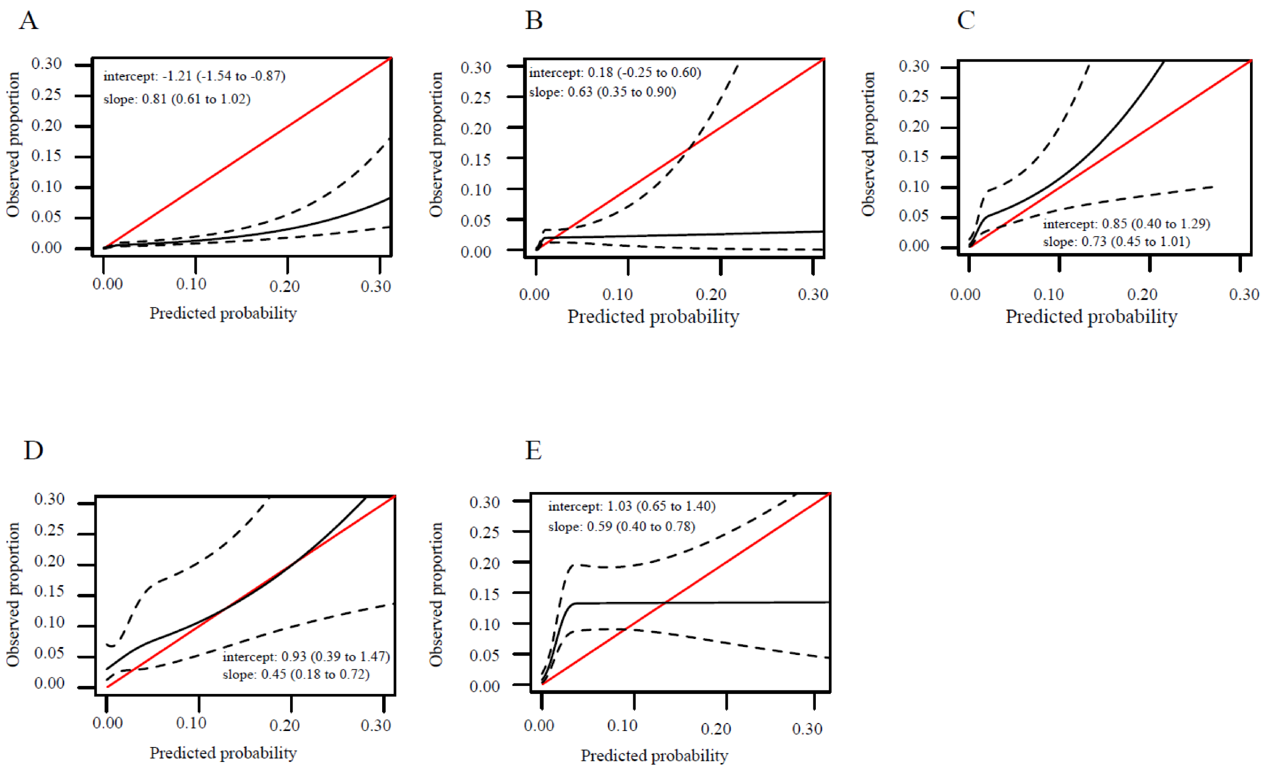

The solid red line with a slope of 1 and intercept of 0 represents ideal prediction accuracy. The dotted lines indicate the 95% confidence interval.

A, Model developed on leave-out EDs with <10 cases, validated on EDs with <10 cases
B, Model developed on leave-out Ljubljana (Slovenia), validated on Ljubljana (Slovenia)

C, Model developed on leave-out London (UK), validated on London (UK)

D, Model developed on leave-out Nijmegen (the Netherlands), validated on Nijmegen, UMC (the Netherlands)

E, Model developed on leave-out Rotterdam (the Netherlands), validated on Rotterdam (the Netherlands)

Legend: ED, emergency department; UK, united kingdom; UMC, University Medical Centre

Appendix 9: Model 2 – model specification and performance

In model 2 the variable ED with low/high IBI incidence is added to the model.

Model 2 – model specification

Model specification of multivariate logistic model for IBI, model 2 with the addition of variable low/high IBI incidence ED

		Coefficients	OR
	(Intercept)	-6.13	0.00
Feverkidstool	Male	-0.16	0.85
	Age < 1 year*	-2.22	0.11
	Age ≥ 1 year*	0.00	1.00
	Temperature	-0.16	0.85
	Fever duration in days	-0.15	0.86
	Tachypnea	-0.47	0.62
	Tachycardia	0.66	1.94
	Hypoxia	-0.81	0.44
	Prolonged capillary refill	-0.31	0.74
	Increased work of breathing	-0.47	0.62
	Ill appearance	1.18	3.26
	Ln CRP	0.75	2.11
NICE warning signs	Abnormal neurology	1.10	3.01
	Non-blanching rash	1.06	2.89
Chronic condition	Complex chronic condition	1.56	4.78
IBI incidence	ED with high IBI incidence (>2%)	1.98	7.26

*Age <1 year and age ≥ 1 year were calculated linear-piecewise:
The risk of children aged < 1 year was calculated: $\beta_{(age <1 year)} \times$ age in years.
The risk of children age ≥ 1 year was calculated: $\beta_{(age <1 year)} \times 1$ + (age in years-1) × $\beta_{(age \geq 1 \ in\ years)}$.

CRP, C-reactive protein; IBI, invasive bacterial infection; ln, natural log

Model 2 - performance

Discrimination:

Development model 2: C-statistic 0.88 (95%CI 0.85-0.90)

Calibration:

Apparent calibration for model 2 for IBI (addition of variable ED with low IBI incidence (<2%) / ED with high IBI incidence (>=2%)). Risk predictions are calculated on the developed model using all data (n=16268). These risk predictions are calibrated in the two groups: EDs with low IBI incidence (A) and EDs with high IBI incidence (B). ED, emergency department; IBI, invasive bacterial infection

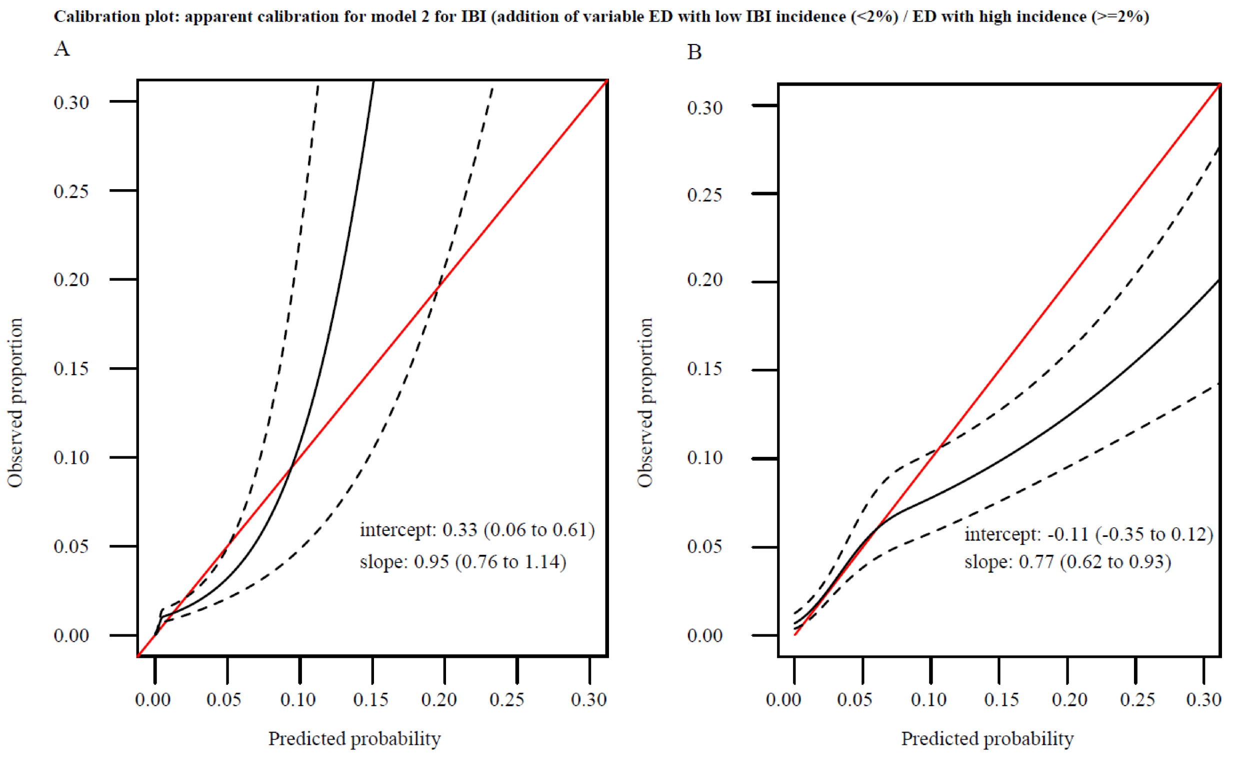

Hagedoorn NN, *et al. Arch Dis Child* 2021; 106:641–647. doi: 10.1136/archdischild-2020-319794

Appendix 10: Performance of the prediction model (model 1)

Decision curve analysis

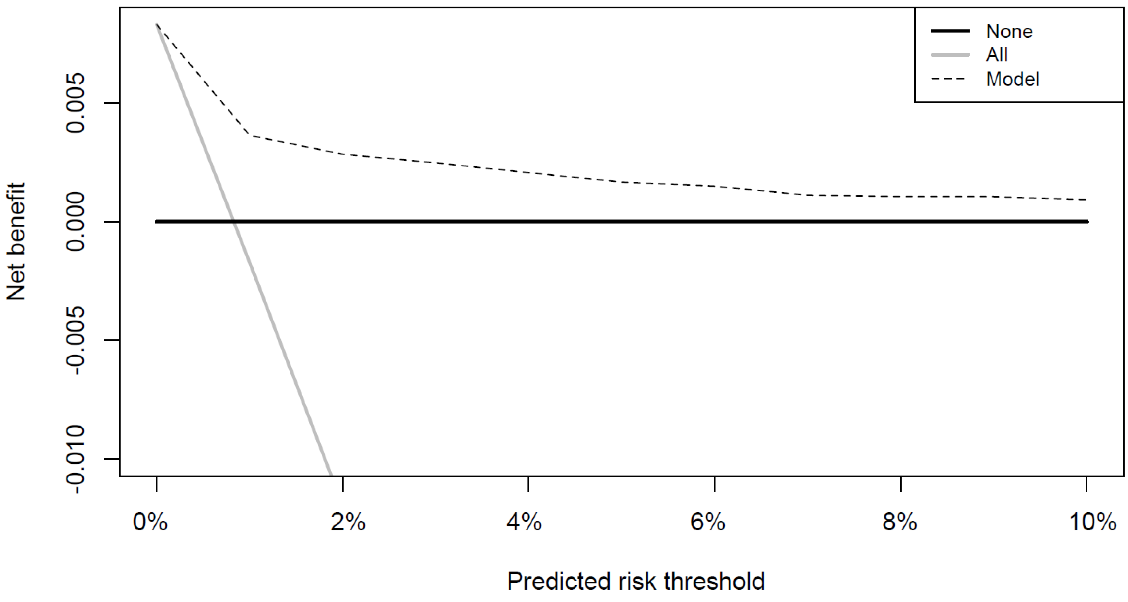

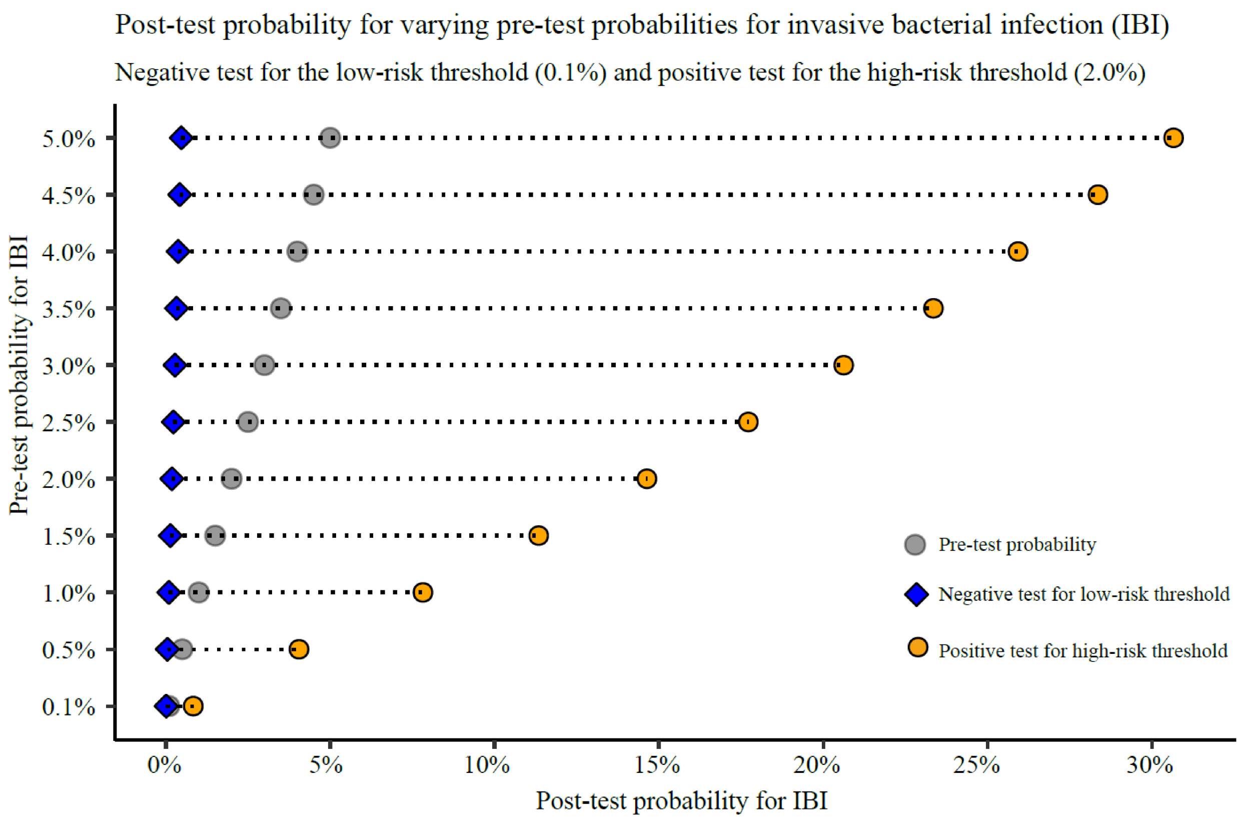

Hagedoorn NN, *et al. Arch Dis Child* 2021; 106:641–647. doi: 10.1136/archdischild-2020-319794

Appendix 11: Sensitivity analysis: model development on population with imputed CRP-level (n=37093)

Model specification of multivariate logistic model for IBI based on population with imputed CRP-level (n=37093)

		Coefficients	OR
	(Intercept)	-9.67	0.00
Feverkidstool	Male	-0.19	0.83
	Age < 1 year*	-2.58	0.08
	Age >1 year*	0.00	1.00
	Temperature	-0.05	0.95
	Fever duration in days	-0.15	0.86
	Tachypnea	-0.43	0.65
	Tachycardia	0.71	2.03
	Hypoxia	-0.86	0.42
	Prolonged capillary refill	0.02	1.02
	Increased work of breathing	-0.34	0.71
	Ill appearance	0.94	2.55
	Ln CRP	0.78	2.17
NICE warning signs	Abnormal neurology	1.54	4.66
	Non-blanching rash	1.40	4.04
Comorbidity	Complex chronic condition	2.43	11.3

*The risk of children aged < 1 year was calculated: $\beta_{(age <1 year)}$×age in years. The risk of children aged < 1 year was calculated: β(age <1 year)×age in years.
The risk of children aged >1 years was calculated with: β(age <1 year)×1+β(age ≥1 year)×(age in years−1).

CRP, C-reactive protein; ln, natural log

Appendix 12: Clinical case examples

Case 1:
A previously healthy, 4 year old boy presents with fever since 1.5 day.
At the ED he has a temperature of 38.9 degrees, heart rate of 160/min, respiratory rate of 45/min, oxygen saturation of 99% and normal capillary refill time. He is ill-appearing, has increased work of breathing and a normal neurological exam.
CRP-level = 10 mg/L.

Risk-prediction:
The patient is at intermediate-risk (>0.1% and <2%) for an invasive bacterial infection.

Case 2:
A previously healthy neonate of 2 months presents with fever since 12 hours.
She has temperature of 38.8 degrees, heart rate of 170/min, respiratory rate of 35/min, normal oxygen saturation and normal capillary refill time. She is ill-appearing and has no increased work of breathing. Neurological exam is normal.
CRP-level = 5 mg/L.

Risk-prediction:
The patient is at high-risk (>2%) for an invasive bacterial infection.

Hagedoorn NN, *et al. Arch Dis Child* 2021; 106:641–647. doi: 10.1136/archdischild-2020-319794

References

References

1. Wolfe I, Cass H, Thompson MJ*, et al.* Improving child health services in the UK: insights from Europe and their implications for the NHS reforms. *Bmj*. 2011;**342**:d1277 Online.
2. Liu L, Oza S, Hogan D*, et al.* Global, regional, and national causes of child mortality in 2000-13, with projections to inform post-2015 priorities: an updated systematic analysis. *Lancet*. 2015;**385**:430-40 Online.
3. Pruitt CM, Neuman MI, Shah SS*, et al.* Factors Associated with Adverse Outcomes among Febrile Young Infants with Invasive Bacterial Infections. *J Pediatr*. 2019;**204**:177-82 e1 Online.
4. van de Maat J, van de Voort E, Mintegi S*, et al.* Antibiotic prescription for febrile children in European emergency departments: a cross-sectional, observational study. *Lancet Infect Dis*. 2019; Online.
5. Herberg JA, Kaforou M, Wright VJ*, et al.* Diagnostic Test Accuracy of a 2-Transcript Host RNA Signature for Discriminating Bacterial vs Viral Infection in Febrile Children. *JAMA*. 2016;**316**:835-45 Online.
6. Oved K, Cohen A, Boico O*, et al.* A novel host-proteome signature for distinguishing between acute bacterial and viral infections. *PLoS One*. 2015;**10**:e0120012 Online.
7. van Houten CB, de Groot JAH, Klein A*, et al.* A host-protein based assay to differentiate between bacterial and viral infections in preschool children (OPPORTUNITY): a double-blind, multicentre, validation study. *Lancet Infect Dis*. 2017;**17**:431-40 Online.
8. Pneumonia Etiology Research for Child Health Study G. Causes of severe pneumonia requiring hospital admission in children without HIV infection from Africa and Asia: the PERCH multi-country case-control study. *Lancet*. 2019; Online.
9. Nijman RG, Vergouwe Y, Thompson M*, et al.* Clinical prediction model to aid emergency doctors managing febrile children at risk of serious bacterial infections: diagnostic study. *BMJ*. 2013;**346**:f1706 Online.
10. Advanced Life Support Group. Advanced Paediatric Life Support: The Practical Approach. 5 ed: Wiley; 2011.
11. The National Institute for Health and Care Excellence (NICE). Fever in under 5s: assessment and initial managment CG160 May 2013. 2013.
12. Simon TD, Cawthon ML, Stanford S*, et al.* Pediatric medical complexity algorithm: a new method to stratify children by medical complexity. *Pediatrics*. 2014;**133**:e1647-54 Online.
13. Tibshirani R. Regression Shrinkage and Selection Via the Lasso. *Journal of the Royal Statistical Society: Series B (Methodological)*. 1996;**58**:267-88 doi: 10.1111/j.2517-6161.1996.tb02080.x [published Online.
14. Steyerberg EW, Harrell FE, Jr. Prediction models need appropriate internal, internal-external, and external validation. *J Clin Epidemiol*. 2016;**69**:245-7 Online.
15. Vos-Kerkhof E, Gomez B, Milcent K*, et al.* Clinical prediction models for young febrile infants at the emergency department: an international validation study. *Arch Dis Child*. 2018;**103**:1033-41 Online.
16. Van Calster B, Nieboer D, Vergouwe Y, De Cock B, Pencina MJ, Steyerberg EW. A calibration hierarchy for risk models was defined: from utopia to empirical data. *J Clin Epidemiol*. 2016;**74**:167-76 Online.
17. Van den Bruel A, Haj-Hassan T, Thompson M, Buntinx F, Mant D, European Research Network on Recognising Serious Infection i. Diagnostic value of clinical features at presentation to identify serious infection in children in developed countries: a systematic review. *Lancet*. 2010;**375**:834-45 Online.
18. Donders AR, van der Heijden GJ, Stijnen T, Moons KG. Review: a gentle introduction to imputation of missing values. *J Clin Epidemiol*. 2006;**59**:1087-91 Online.
19. Zhao Y, Long Q. Variable selection in the presence of missing data: imputation-based methods. *WIREs Computational Statistics*. 2017;**9**:e1402 doi: 10.1002/wics.1402 [published Online.
20. Su TL, Jaki T, Hickey GL, Buchan I, Sperrin M. A review of statistical updating methods for clinical prediction models. *Stat Methods Med Res*. 2018;**27**:185-97 Online.

21. Steyerberg EW, Borsboom GJ, van Houwelingen HC, Eijkemans MJ, Habbema JD. Validation and updating of predictive logistic regression models: a study on sample size and shrinkage. *Stat Med*. 2004;**23**:2567-86 Online.
22. Friedman J, Hastie T, Tibshirani R. Regularization Paths for Generalized Linear Models via Coordinate Descent. *J Stat Softw*. 2010;**33**:1-22 Online.

Version: 6.1 September 2020

<u>PARTNER: IMPERIAL COLLEGE (UK)</u>
<u>Chief investigator/PERFORM coordinator:</u>

Michael Levin

<u>Principal and co-investigators; work package leads (alphabetical order)</u>

Aubrey Cunnington (grant application)

Tisham De (work package lead)

Jethro Herberg (Principle Investigator, Deputy Coordinator, grant application)

Myrsini Kaforou (grant application, work package lead)

Victoria Wright (grant application, Scientific Coordinator)

<u>Research Group (alphabetical order)</u>

Lucas Baumard; Evangelos Bellos; Giselle D'Souza; Rachel Galassini; Dominic Habgood-Coote; Shea Hamilton; Clive Hoggart; Sara Hourmat; Heather Jackson; Ian Maconochie; Stephanie Menikou; Naomi Lin; Samuel Nichols; Ruud Nijman; Ivonne Pena Paz; Priyen Shah; Ching-Fen Shen; Ortensia Vito; Clare Wilson

<u>Clinical recruitment at Imperial College Healthcare NHS Trust (alphabetical order))</u>

Amina Abdulla; Ladan Ali; Sarah Darnell; Rikke Jorgensen; Sobia Mustafa; Salina Persand

<u>Imperial College Faculty of Engineering</u>

Molly Stevens (co-investigator), Eunjung Kim (research group); Benjamin Pierce (research group)

Version: 6.1 September 2020

Clinical recruitment at Brighton and Sussex University Hospitals

Katy Fidler (Principle Investigator)

Julia Dudley (Clinical Research Registrar)

Research nurses: Vivien Richmond, Emma Tavliavini

Clinical recruitment at National Cheng Kung University Hospital

Ching-Fen Shen (Principal Investigator); Ching-Chuan Liu (Co-investigator); Shih-Min Wang (Co-investigator), funded by the Center of Clinical Medicine Research, National Cheng Kung University

SERGAS Partner (Spain)

Principal Investigators

Federico Martinón-Torres[1]

Antonio Salas[1,2]

GENVIP RESEARCH GROUP (in alphabetical order):

Fernando Álvez González[1], Cristina Balo Farto[1], Ruth Barral-Arca[1,2], María Barreiro Castro[1], Xabier Bello[1,2], Mirian Ben García[1], Sandra Carnota[1], Miriam Cebey-López[1], María José Curras-Tuala[1,2], Carlos Durán Suárez[1], Luisa García Vicente[1], Alberto Gómez-Carballa[1,2], Jose Gómez Rial[1], Pilar Leboráns Iglesias[1], Federico Martinón-Torres[1], Nazareth Martinón-Torres[1], José María Martinón Sánchez[1], Belén Mosquera Pérez[1], Jacobo Pardo-Seco[1,2], Lidia Piñeiro Rodríguez[1], Sara Pischedda[1,2], Sara Rey Vázquez[1], Irene Rivero Calle[1], Carmen Rodríguez-Tenreiro[1], Lorenzo Redondo-Collazo[1], Miguel Sadiki Ora[1], Antonio Salas[1,2], Sonia Serén Fernández[1], Cristina Serén Trasorras[1], Marisol Vilas Iglesias[1].

[1] Translational Pediatrics and Infectious Diseases, Pediatrics Department, Hospital Clínico Universitario de Santiago, Santiago de Compostela, Spain, and GENVIP Research Group (www.genvip.org), Instituto de Investigación Sanitaria de Santiago, Universidad de Santiago de Compostela, Galicia, Spain.

[2] Unidade de Xenética, Departamento de Anatomía Patolóxica e Ciencias Forenses, Instituto de Ciencias Forenses, Facultade de Medicina, Universidade de Santiago de Compostela, and GenPop Research Group, Instituto de Investigaciones Sanitarias (IDIS), Hospital Clínico Universitario de Santiago, Galicia, Spain

[3] Fundación Pública Galega de Medicina Xenómica, Servizo Galego de Saúde (SERGAS), Instituto de Investigaciones Sanitarias (IDIS), and Grupo de Medicina Xenómica, Centro de Investigación Biomédica en Red de Enfermedades Raras (CIBERER), Universidade de Santiago de Compostela (USC), Santiago de Compostela, Spain

RSU Partner (Latvia)

Principal Investigator

Dace Zavadska[1,2]

Other RSU group authors (in alphabetical order):

Anda Balode[1,2], Arta Bārzdiņa[1,2], Dārta Deksne[1,2], Dace Gardovska[1,2], Dagne Grāvele[2], Ilze Grope[1,2], Anija Meiere[1,2], Ieva Nokalna[1,2], Jana Pavāre[1,2], Zanda Pučuka[1,2], Katrīna Selecka[1,2], Aleksandra Sidorova[1,2], Dace Svile[2], Urzula Nora Urbāne[1,2].

[1] Riga Stradins university, Riga, Latvia.

[2] Children clinical university hospital, Riga, Latvia.

Medical Research Council Unit The Gambia (MRCG) at LSHTM Partner

Principal Investigator

Effua Usuf

Additional Investigators

Kalifa Bojang
Syed M. A. Zaman
Fatou Secka
Suzanne Anderson
Anna Rocalsatou Sarr
Momodou Saidykhan
Saffiatou Darboe
Samba Ceesay
Umberto D’alessandro

Medical Research Council Unit The Gambia at LSHTM
P O Box 273,

Version: 6.1 September 2020

Fajara, The Gambia

ERASMUS MC-Sophia Children's Hospital

Principal Investigator

Henriëtte A. Moll[1]

Research group

Dorine M. Borensztajn[1], Nienke N. Hagedoorn, Chantal Tan [1], [1], Clementien L. Vermont[2], Joany Zachariasse [1]

Additional investigator

W Dik [3]

[1] Erasmus MC-Sophia Children's Hospital, Department of General Paediatrics, Rotterdam, the Netherlands

[2] Erasmus MC-Sophia Children's Hospital, Department of Paediatric Infectious Diseases & Immunology, Rotterdam, the Netherlands

[3] Erasmus MC, Department of immunology, Rotterdam, the Netherlands

Swiss Pediatric Sepsis Study

Principal Investigators*:*

Philipp Agyeman, MD [1] (ORCID 0000-0002-8339-5444), Luregn J Schlapbach, MD, FCICM [2,3] (ORCID 0000-0003-2281-2598)

Clinical recruitment at University Children's Hospital Bern for PERFORM:

Christoph Aebi [1], Verena Wyss [1], Mariama Usman [1]

Principal and co-investigators for the Swiss Pediatric Sepsis Study:

Philipp Agyeman, MD [1], Luregn J Schlapbach, MD, FCICM [2,3], Eric Giannoni, MD [4,5], Martin Stocker, MD [6], Klara M Posfay-Barbe, MD [7], Ulrich Heininger, MD [8], Sara Bernhard-Stirnemann, MD [9], Anita Niederer-Loher, MD [10], Christian Kahlert, MD [10], Giancarlo Natalucci, MD [11], Christa Relly, MD [12], Thomas Riedel, MD [13], Christoph Aebi, MD [1], Christoph Berger, MD [12] **for the Swiss Pediatric Sepsis Study**

Affiliations:

[1] Department of Pediatrics, Inselspital, Bern University Hospital, University of Bern, Switzerland

[2] Neonatal and Pediatric Intensive Care Unit, Children's Research Center, University Children's Hospital Zurich, University of Zurich, Zurich, Switzerland

[3]Child Health Research Centre, University of Queensland, and Queensland Children`s Hospital, Brisbane, Australia

[4] Clinic of Neonatology, Department Mother-Woman-Child, Lausanne University Hospital and University of Lausanne, Switzerland

[5] Infectious Diseases Service, Department of Medicine, Lausanne University Hospital and University of Lausanne, Switzerland

[6] Department of Pediatrics, Children's Hospital Lucerne, Lucerne, Switzerland

[7] Pediatric Infectious Diseases Unit, Children's Hospital of Geneva, University Hospitals of Geneva, Geneva, Switzerland

[8] Infectious Diseases and Vaccinology, University of Basel Children's Hospital, Basel, Switzerland

[9] Children's Hospital Aarau, Aarau, Switzerland

[10] Division of Infectious Diseases and Hospital Epidemiology, Children's Hospital of Eastern Switzerland St. Gallen, St. Gallen, Switzerland

[11] Department of Neonatology, University Hospital Zurich, Zurich, Switzerland

[12] Division of Infectious Diseases and Hospital Epidemiology, and Children's Research Center, University Children's Hospital Zurich, Switzerland

[13] Children's Hospital Chur, Chur, Switzerland

Liverpool Partner

<u>Principal Investigators</u>

Enitan D Carrol[1,2,3]

Stéphane Paulus [1,]

<u>Research Group (in alphabetical order):</u>

Elizabeth Cocklin[1], Rebecca Jennings[4], Joanne Johnston[4], Simon Leigh[1], Karen Newall[4], Sam Romaine[1]

Version: 6.1 September 2020

[1] Department of Clinical Infection, Microbiology and Immunology, University of Liverpool Institute of Infection and Global Health , Liverpool, England

[2] Alder Hey Children's Hospital, Department of Infectious Diseases, Eaton Road, Liverpool, L12 2AP

[3] Liverpool Health Partners, 1st Floor, Liverpool Science Park, 131 Mount Pleasant, Liverpool, L3 5TF

[4]Alder Hey Children's Hospital, Clinical Research Business Unit, Eaton Road, Liverpool, L12 2AP

NKUA Partner (Greece)

Principal investigator: Professor **Maria Tsolia** (all activities)

Investigator/Research fellow: **Irini Eleftheriou** (all activities)

Additional investigators:

Recruitment: Maria Tambouratzi

Lab: Antonis Marmarinos (Quality Manager)

Lab: Marietta Xagorari

Kelly Syggelou

2nd Department of Pediatrics, National and Kapodistrian University of Athens,

"P. and A. Kyriakou" Children's Hospital

Thivon and Levadias

Goudi, Athens

Micropathology Ltd :

Principal Investigator:

Professor Colin Fink[1], Clinical Microbiologist

Additional investigators

Dr Marie Voice[1], Post doc scientist

Version: 6.1 September 2020

Dr. Leo Calvo-Bado[1], Post doc scientist

[1] Micropathology Ltd, The Venture Center, University of Warwick Science Park, Sir William Lyons Road, Coventry, CV4 7EZ.

Medical University of Graz, Austria (MUG)

Principal Investigator:

Werner Zenz[1] (all activities)

Co-investigators (in alphabetical order)

Benno Kohlmaier[1] (all activities)

Nina A. Schweintzger[1] (all activities)

Manfred G. Sagmeister[1] (study design, consortium wide sample management)

Research team

Daniela S. Kohlfürst[1] (study design)

Christoph Zurl[1] (BIVA PIC)

Alexander Binder[1] (grant application)

Recruitment team, data managers, (in alphabetical order):

Susanne Hösele[1], Manuel Leitner[1], Lena Pölz[1], Glorija Rajic[1],

Clinical recruitment partners (in alphabetical order):

Sebastian Bauchinger[1], Hinrich Baumgart[4], Martin Benesch[3], Astrid Ceolotto[1], Ernst Eber[2], Siegfried Gallistl[1], Gunther Gores[5], Harald Haidl[1], Almuthe Hauer[1], Christa Hude[1], Markus Keldorfer[5], Larissa Krenn[4], Heidemarie Pilch[5], Andreas Pfleger[2], Klaus Pfurtscheller[4], Gudrun

Nordberg[5], Tobias Niedrist[8], Siegfried Rödl[4], Andrea Skrabl-Baumgartner[1], Matthias Sperl[7], Laura Stampfer[5], Volker Strenger[3], Holger Till[6], Andreas Trobisch[5], Sabine Löffler[5]

Author Affiliations:

[1] Department of Pediatrics and Adolescent Medicine, Division of General Pediatrics, Medical University of Graz, Graz, Austria

[2]Department of Pediatric Pulmonology, Medical University of Graz, Graz, Austria

[3]Department of Pediatric Hematooncoloy, Medical University of Graz, Graz, Austria

[4]Paediatric Intensive Care Unit, Medical University of Graz, Graz, Austria

[5]University Clinic of Paediatrics and Adolescent Medicine Graz, Medical University Graz, Graz,Austria

[6]Department of Paediatric and Adolescence Surgery, Medical University Graz, Graz, Austria

[7]Department of Pediatric Orthopedics, Medical University Graz, Graz, Austria

[8]Clinical Institute of Medical and Chemical Laboratory Diagnostics, Medical University Graz, Graz, Austria

London School of Hygiene and Tropical Medicine

WP 1 WP2, WP5

Principal Investigator:

Dr Shunmay Yeung[1,2 3] PhD, MBBS, FRCPCH, MRCP, DTM&H

Research Group

Dr Juan Emmanuel Dewez[1] MD, DTM&H, MSc

Prof Martin Hibberd [1] BSc, PhD

Mr David Bath[2] MSc, MAppFin, BA(Hons)

Dr Alec Miners[2] BA(Hons), MSc, PhD

Dr Ruud Nijman[3] PhD MSc MD MRCPCH

Dr Catherine Wedderburn[1] BA, MBChB, DTM&H, MSc, MRCPCH

Version: 6.1 September 2020

Ms Anne Meierford[1] MSc, BMedSc, BMBS

Dr Baptiste Leurent[4], PhD, MSc

1. Faculty of Infectious and Tropical Disease, London School of Hygiene and Tropical Medicine, London, UK
2. Faculty of Public Health and Policy, London School of Hygiene and Tropical Medicine, London, UK
3. Department of Paediatrics, St. Mary's Hospital Imperial College Hospital, London, UK
4. Faculty of Epidemiology and Population Health, London School of Hygiene and Tropical Medicine, London, UK

Radboud University Medical Center (RUMC), The Netherlands

Principal Investigators:
Ronald de Groot [1], Michiel van der Flier [1,2,3], Marien I. de Jonge[1]

Co-investigators Radboud University Medical Center (in alphabetical order):
Koen van Aerde[1,2], Wynand Alkema[1], Bryan van den Broek[1], Jolein Gloerich[1], Alain J. van Gool[1], Stefanie Henriet[1,2], Martijn Huijnen[1], Ria Philipsen[1], Esther Willems[1]

Investigators PeDBIG PERFORM DUTCH CLINICAL NETWORK (in alphabetical order):
G.P.J.M. Gerrits[8], M. van Leur[8,] J. Heidema [4],L. de Haan[1,2] C.J. Miedema [5], C. Neeleman [1] C.C. Obihara [6], G.A. Tramper-Stranders7[6]

1. Radboud University Medical Center, Nijmegen, The Netherlands
2. Amalia Children's Hospital, Nijmegen, The Netherlands
3. Wilhelmina Children's Hospital, University Medical Center Utrecht, Utrecht, The Netherlands
4. St. Antonius Hospital, Nieuwegein, The Netherlands
5. Catharina Hospital, Eindhoven, The Netherlands
6. ETZ Elisabeth, Tilburg, The Netherlands
7. Franciscus Gasthuis, Rotterdam, The Netherlands
8. Canisius Wilhelmina Hospital, Nijmegen, The Netherlands

Oxford team (UK)

Hagedoorn NN, *et al. Arch Dis Child* 2021; 106:641–647. doi: 10.1136/archdischild-2020-319794

Version: 6.1 September 2020

Principal Investigators

Andrew J. Pollard[1,2], Rama Kandasamy[1,2], Stéphane Paulus [1,2]

Additional Investigators

Michael J. Carter[1,2], Daniel O'Connor[1,2], Sagida Bibi[1,2], Dominic F. Kelly[1,2], Meeru Gurung[3], Stephen Thorson[3], Imran Ansari[3], David R. Murdoch[4], Shrijana Shrestha[3], Zoe Oliver[5]

Author Affiliations:

[1]Oxford Vaccine Group, Department of Paediatrics, University of Oxford, Oxford, United Kingdom.

[2]NIHR Oxford Biomedical Research Centre, Oxford, United Kingdom.

[3]Paediatric Research Unit, Patan Academy of Health Sciences, Kathmandu, Nepal.

[4]Department of Pathology, University of Otago, Christchurch, New Zealand.

[5] Department of Paediatrics, University of Oxford.

Newcastle University, Newcastle upon Tyne, (UK)

Principal Investigator:

Marieke Emonts [1,2,3] (all activities)

Co-investigators

Emma Lim[2,3,7] (all activities)

Lucille Valentine[4]

Recruitment team (alphabetical), data-managers, and GNCH Research unit:

10

Version: 6.1 September 2020

Karen Allen[5], Kathryn Bell[5], Adora Chan[5], Stephen Crulley[5], Kirsty Devine[5], Daniel Fabian[5], Sharon King[5], Paul McAlinden[5], Sam McDonald[5], Anne McDonnell2,[5], Ailsa Pickering[2,5], Evelyn Thomson[5], Amanda Wood[5], Diane Wallia[5], Phil Woodsford[5],

Sample processing: Frances Baxter[5], Ashley Bell[5], Mathew Rhodes[5]

PICU recruitment

Rachel Agbeko[8]

Christine Mackerness[8]

Students MOFICHE

Bryan Baas[2], Lieke Kloosterhuis[2], Wilma Oosthoek[2]

Students/medical staff PERFORM

Tasnim Arif[6], Joshua Bennet[2], Kalvin Collings[2], Ilona van der Giessen[2], Alex Martin[2], Aqeela Rashid[6], Emily Rowlands[2], Gabriella de Vries[2], Fabian van der Velden[2]

Engagement work/ethics/cost effectiveness

Lucille Valentine [4], Mike Martin[9], Ravi Mistry[2], Lucille Valentine[4]

Author Affiliations:

[1] Translational and Clinical Research Institute, Newcastle University, Newcastle upon Tyne UK

[2]Great North Children's Hospital, Paediatric Immunology, Infectious Diseases & Allergy, Newcastle upon Tyne Hospitals NHS Foundation Trust, Newcastle upon Tyne, United Kingdom.

[3]NIHR Newcastle Biomedical Research Centre based at Newcastle upon Tyne Hospitals NHS Trust and Newcastle University, Westgate Rd, Newcastle upon Tyne NE4 5PL, United Kingdom

[4]Newcastle University Business School, Centre for Knowledge, Innovation, Technology and Enterprise (KITE), Newcastle upon Tyne, United Kingdom

[5]Great North Children's Hospital, Research Unit, Newcastle upon Tyne Hospitals NHS Foundation Trust, Newcastle upon Tyne, United Kingdom.

[6]Great North Children's Hospital, Paediatric Oncology, Newcastle upon Tyne Hospitals NHS Foundation Trust, Newcastle upon Tyne, United Kingdom.

Version: 6.1 September 2020

[7]Population Health Sciences Institute, Newcastle University, Newcastle upon Tyne, UK

[8]Great North Children's Hospital, Paediatric Intensive Care Unit, Newcastle upon Tyne Hospitals NHS Foundation Trust, Newcastle upon Tyne, United Kingdom.

[9]Northumbria University, Newcastle upon Tyne, United Kingdom.

LMU Munich Partner (Germany)

Principal Investigator:

Ulrich von Both[1,2] MD, FRCPCH (all activities)

Research group:

Laura Kolberg[1] MSc (all activities)

Manuela Zwerenz[1] MSc, Judith Buschbeck[1] PhD

Clinical recruitment partners (in alphabetical order):

Christoph Bidlingmaier[3], Vera Binder[4], Katharina Danhauser[5], Nikolaus Haas[10], Matthias Griese[6], Tobias Feuchtinger[4], Julia Keil[9], Matthias Kappler[6], Eberhard Lurz[7], Georg Muench[8], Karl Reiter[9], Carola Schoen[9]

Author Affiliations:

[1]Div. Paediatric Infectious Diseases, Hauner Children's Hospital, University Hospital, Ludwig Maximilians University (LMU), Munich, Germany

[2]German Center for Infection Research (DZIF), Partner Site Munich, Munich, Germany

[3]Div. of General Paediatrics, [4]Div. Paediatric Haematology & Oncology, [5]Div. of Paediatric Rheumatology, [6]Div. of Paediatric Pulmonology, [7]Div. of Paediatric Gastroenterology, [8]Neonatal Intensive Care Unit, [9]Paediatric Intensive Care Unit Hauner Children's Hospital, University

Version: 6.1 September 2020

Hospital, Ludwig Maximilians University (LMU), Munich, Germany, [10]Department Pediatric Cardiology and Pediatric Intensive Care, University Hospital, Ludwig Maximilians University (LMU), Munich, Germany

bioMérieux, France

Principal Investigator:
François Mallet[1,2, 3]

Research Group:
Karen Brengel-Pesce[1,2, 3]
Alexandre Pachot[1]
Marine Mommert[1,2]

[1]Open Innovation & Partnerships (OIP), bioMérieux S.A., Marcy l'Etoile, France
[2] Joint research unit Hospice Civils de Lyon - bioMérieux, Centre Hospitalier Lyon Sud, 165 Chemin du Grand Revoyet, 69310 Pierre-Bénite, France
[3] EA 7426 Pathophysiology of Injury-induced Immunosuppression, University of Lyon1-Hospices Civils de Lyon-bioMérieux, Hôpital Edouard Herriot, 5 Place d'Arsonval, 69437 Lyon Cedex 3, France

Department of Infectious Diseases, University Medical Centre Ljubljana, Slovenia

Principal Investigator:

Marko Pokorn[1,2,3] MD, PhD

Research Group:

Mojca Kolnik[1] MD, Katarina Vincek[1] MD, Tina Plankar Srovin[1] MD, PhD, Natalija Bahovec[1] MD, Petra Prunk[1] MD, Veronika Osterman[1] MD, Tanja Avramoska[1] MD

Affiliations:

[1]Department of Infectious Diseases, University Medical Centre Ljubljana, Japljeva 2, SI-1525 Ljubljana, Slovenia

[2]University Childrens' Hospital, University Medical Centre Ljubljana, Ljubljana, Slovenia

[3]Department of Infectious Diseases and Epidemiology, Faculty of Medicine, University of Ljubljana, Slovenia

Version: 6.1 September 2020

Amsterdam, Academic Medical Hospital & Sanquin Research Institute (NL)

Principal Investigator:

Taco Kuijpers [1,2]

Co-investigators

Ilse Jongerius [2]

Recruitment team (EUCLIDS, PERFORM):

J.M. van den Berg[1], D. Schonenberg[1], A.M. Barendregt[1], D. Pajkrt[1], M. van der Kuip[1,3], A.M. van Furth[1,3]

Students PERFORM

Evelien Sprenkeler [2], Judith Zandstra [2],

Technical support PERFORM

G. van Mierlo [2], J. Geissler [2]

Author Affiliations:

[1] Amsterdam University Medical Center (Amsterdam UMC), location Academic Medical Center (AMC), Dept of Pediatric Immunology, Rheumatology and Infectious Diseases, University of Amsterdam, Amsterdam, the Netherlands

[2] Sanquin Research Institute, & Landsteiner Laboratory at the AMC, University of Amsterdam, Amsterdam, the Netherlands.

[3] Amsterdam University Medical Center (Amsterdam UMC), location Vrije Universiteit Medical Center (VUMC), Dept of Pediatric Infectious Diseases and Immunology, Free University (VU), Amsterdam, the Netherlands (former affiliation)
